# MAGI3 enhances sensitivity to sunitinib in renal cell carcinoma by suppressing the MAS/ERK axis and serves as a prognostic marker

**DOI:** 10.1038/s41419-025-07427-0

**Published:** 2025-02-16

**Authors:** Haibo Wang, Yibin Chen, Ying Yang, Ran Song, Siyu Gu, Xuedi Cao, Lijie Zhang, Yang Yang, Tianzhong Hou, Xuan Qi, Yumeng Yang, Yue Wang, Tao Bai, Duiping Feng, Xiaomei Yang, Junqi He

**Affiliations:** 1 Beijing Key Laboratory for Tumor Invasion and Metastasis, Department of Biochemistry and Molecular Biology, Capital Medical University, Beijing, China; 2 Laboratory for Clinical Medicine, Capital Medical University, Beijing, China; 3 Beijing Laboratory of Oral Health, Capital Medical University, Beijing, China; 4 Core Facilities Center, Capital Medical University, Beijing, China; 5 Center for Endocrine Metabolism and Immune Diseases, Beijing Luhe Hospital, Capital Medical University, Beijing, China; 6 Department of Neurobiology, School of Basic Medical Sciences, Capital Medical University, Beijing, China; 7 Department of Pathology, First Hospital of Shanxi Medical University, Taiyuan, China; 8 Department of Interventional Radiology, First Hospital of Shanxi Medical University, Taiyuan, China

**Keywords:** Tumour-suppressor proteins, Targeted therapies, G protein-coupled receptors

## Abstract

Clear cell renal cell carcinoma (ccRCC) exhibits considerable heterogeneity, with approximately 25% of localized cases susceptible to relapse, highlighting the challenge of the absence of reliable predictive biomarkers for personalized treatment. Meanwhile, metastatic renal cell carcinoma is characterized by unfavorable survival rates, and although Sunitinib offers partial benefits, the clinical advantages are often constrained by drug resistance and adverse side effects. Here, MAGI3 was associate with ccRCC progression, as identified through comprehensive bioinformatics analysis of clinical datasets. A low level of MAGI3 emerged as a high-risk factor for ccRCC, indicating its potential as a prognostic marker. Individuals with MAGI3 expression in middle-to-low levels displayed a significantly poorer survival rate, indicating a need for additional treatment even in the early stages of ccRCC. Furthermore, patients with MAGI3 expression in middle-to-high levels exhibited increased sensitivity to Sunitinib compared to those with lower MAGI3 levels, suggesting that individuals with MAGI3 expression at middle levels may potentially benefit from Sunitinib treatment even in the early stages of ccRCC. Through its interaction with the MAS receptor, MAGI3 has been identified as a regulator of cell proliferation and a determinant of Sunitinib resistance in ccRCC, operating via the Ang-(1-7)/MAS/ERK axis. The loss of MAGI3 expression in ccRCC patients activated the ERK signaling pathway, contributing to both cancer progression and Sunitinib resistance. Therefore, our study not only highlight MAGI3’s pivotal role in ccRCC progression and Sunitinib resistance, but also reinforces MAGI3’s prospective value as a predictive marker.

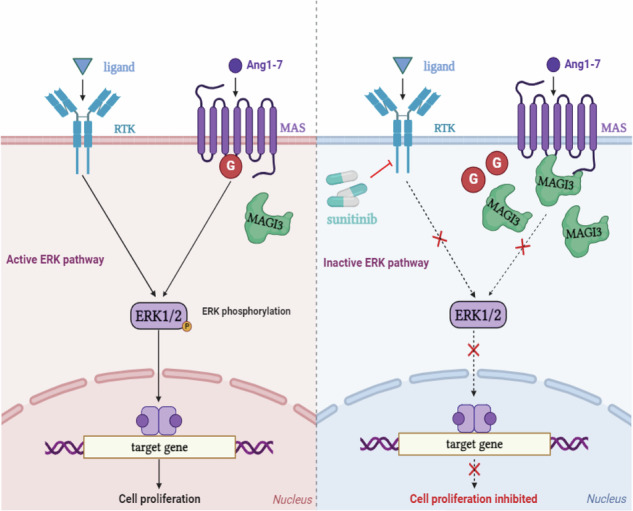

## Introduction

Renal cell carcinoma (RCC) is a highly lethal urological malignancy, with clear cell renal cell carcinoma (ccRCC) being the most prevalent subtype, and its incidence steadily rising in recent years [1, 2]. The survival rates exhibit significantly difference between early-stage or localized RCC and metastatic RCC (mRCC), with localized RCC exhibiting a generally favorable rate, while mRCC falls within a range of 5–12% in a 5-year period [3, 4]. Surgical nephrectomy stands as a highly effective treatment for early-stage or localized ccRCC. Traditional therapeutic strategies generally do not incorporate additional adjuvant therapies, especially for individuals at low risk or in early-stage cases, such as stage I. Despite its effectiveness, the substantial heterogeneity within ccRCC leads to diverse outcomes, even among patients in the same stage, complicating treatment and management. Notably, about 25% of patients with localized ccRCC experience relapse following local therapy [5, 6]. Nevertheless, the absence of reliable biomarkers presents a challenge in accurately identifying high-risk localized ccRCC patients for stratification and personalized management.

mRCC is a life-threatening disease characterized by poor overall survival (OS) rates. Despite advancements in diagnostic techniques, approximately 20–30% of ccRCC patients present with local or distant metastases at the time of diagnosis [7]. The current therapeutic approaches demonstrate limited efficacy, mainly attributed to mRCC’s intrinsic resistance to chemotherapy or radiotherapy [8]. Cytokine treatment benefits only a small subset of patients, yielding a discouraging 5-year survival rate of just 9% [9, 10].

Recently, several small molecule tyrosine kinase inhibitors (TKIs) have been developed to treat advanced renal cell carcinoma (aRCC) or mRCC [11]. Notably, Sunitinib has proven to be an effective first-line treatment, markedly enhancing both progression-free survival (PFS) and overall survival (OS) in aRCC or mRCC cases [12–14]. Despite its effectiveness, Sunitinib has limitations, with an objective response rate of approximately 30%, and a significant number of patients eventually experiencing relapse due to the development of drug resistance [15, 16].

Multiple lines of evidence indicate that resistance to Sunitinib in ccRCC is driven by various factors, including interactions with the tumor microenvironment, increased tumor invasiveness and metastasis, and resistance mediated by noncoding RNAs [17–19]. Tumors also have the ability to activate alternative pathways that bypass the targeted receptors, promoting cell survival, proliferation, and angiogenesis. Overcoming Sunitinib resistance presents a significant challenge, primarily attributed to the insufficient inhibition of its intended targets. This inadequacy compromises the drug’s efficacy in effectively controlling tumor growth [20]. Sunitinib, a multi-targeted tyrosine kinase inhibitor (TKI), exerts inhibitory effects on VEGFR, PDGFR, and EGFR within tumor cells. Among the downstream pathways activated by these surface receptors, the MAPK/ERK pathway plays a crucial role [21]. This pathway transmits growth and survival signals from receptors to nuclear transcription machinery [22, 23]. Dysregulation of the MAPK/ERK pathway is a common phenomenon in various human cancers, resulting in alterations in gene expression, and promoting cancer cell proliferation, progression and angiogenesis [24, 25]. Recent studies have highlighted the efficacy of inhibiting the MAPK/ERK pathway in inducing apoptosis and impeding metastasis in RCC cells [26]. The anti-tumor effects of Sunitinib primarily result from its ability to inhibit the ERK pathway, leading to increased apoptosis [27–29]. However, resistance to Sunitinib often develops due to insufficient ERK inhibition or excessive activation through alternative pathways that operate independently of growth factor receptors in cancer cells [30]. This phenomenon has been observed in various cancer types, including RCC tumors [31–33]. Nevertheless, the precise molecular mechanisms responsible for dysregulated ERK activation in mRCC remain not completely understood.

In this study, an analysis of differentially expressed genes (DEGs) between tumor and normal renal tissues revealed the significant enrichment of the MAPK/ERK cascade within the DEGs. Among the subset of genes related to the MAPK pathway, MAGI3 expression correlates with well-prognostic ccRCC, even at an early stage. Through its interaction with the MAS receptor, MAGI3 was identified as a regulator of cell proliferation and a determinant of Sunitinib resistance in ccRCC, operating via the Ang-(1-7)/MAS/ERK axis. Loss of MAGI3 expression in ccRCC patients activated the ERK signaling pathway, contributing to both cancer progression and Sunitinib resistance. These findings highlight MAGI3 as a novel tumor suppressor in ccRCC and indicate the importance of the ACE2/Ang-(1-7)/MAS axis in Sunitinib resistance in ccRCC. Additionally, this research highlights the potential of MAGI3 as a predictive marker, offering promise in tailoring treatments for early-stage ccRCC patients and thereby improving clinical decision-making.

## Materials and methods

### Clear cell renal cell carcinoma specimens

This study retrospectively collected surgical specimens and corresponding data from metastatic ccRCC patients at the First Hospital of Shanxi Medical University (2016–2021). The inclusion criteria were as follows: 1) Patients underwent primary tumor resection, confirming ccRCC with at least one measurable metastatic lesion; 2) Patients received oral Sunitinib (50 mg/day for 4 weeks, followed by a 2-week break) for a 6-week cycle. Computed Tomography scans assessed therapeutic response every two cycles; 3) Complete follow-up information on Sunitinib responsiveness and prognosis. The exclusion criteria were: 1) Other combination therapies; 2) Discontinuation of Sunitinib before one cycle completion due to adverse reactions or compliance issues. The study included 47 patients (33 males, 14 females, average age: 54.2 ± 9.6 years). Tissue microarrays, containing 90 matched pairs of ccRCC and adjacent normal tissues (51 males, 39 females, average age: 59.0 ± 11.5 years) were sourced from Shanghai Outdo Biotech. The research protocol adhered to the Declaration of Helsinki, approved by the Ethics Committee of Capital Medical University and the First Hospital of Shanxi Medical University. All participating patients provided written informed consent for sample use and publication of related data.

### Animal study

To establish a xenograft tumor model, 5 ×10^6^ control vector or MAGI3-overexpressing 786-O cells were subcutaneously injected into the dorsal flank of BALB/c nude mice (male, 6 weeks old, weight 16–18 g, *n* = 18). Tumor growth was monitored, and size was calculated using the formula: 1/2 × length × width^2^. For Sunitinib treatment, 1 ×10^7^ cells were injected, and mice (male, 6 weeks old, weight 16-18 g, *n* = 10) received Sunitinib (i.g. 50 mg/kg/day) and Ang-(1-7) (s.c. 20 ug/kg/day) when tumors reached 80–100 mm^3^.

Experiments concluded when tumors did not exceed 1000 mm^3^; mice were humanely euthanized, tumors dissected, and subjected to fixation, paraffin embedding, and IHC staining.

*Magi3* knockout mice (*Magi3*^−/−^) and their wild-type counterparts (*Magi3*^*+/+*^) were generated by Shanghai Model Organisms Center, Inc., and genotyped using specific primers. Ang-(1-7) or A779 (dissolved in 0.9% saline) was administered intraperitoneally, with doses of 60 ug/kg for Ang-(1-7) and 0.5 ug/kg for A779, given 30 minutes prior. Kidney tissue samples underwent IHC and western blotting, with each experimental group comprising 2 male mice aged 6 weeks and weighing between 16 and 20 g.

Mice were randomly allocated to either the control or experimental groups using a random number method. Grouping was conducted without utilizing a blind method, and sample sizes were determined based on previous experimental experience to ensure that statistical differences could be achieved.

All animal experiments were conducted in compliance with the National Institutes of Health guidelines for the Care and Use of Laboratory Animals and approved by the Animal Use and Care Committee of Capital Medical University (approval numbers AEEI-2020-133 and AEEI-2018-16). Our study adhered to the guidelines set forth by our ethics committee, which approved a maximal tumor size/burden of 1000 mm^3^. We confirm that throughout the study, the maximal tumor size/burden limit was strictly adhered to and not exceeded.

### Statistical analysis

Statistical analysis utilized GraphPad Prism 7 software. Data, unless specified otherwise, is presented as mean ± SEM. Measurement data were assessed for normal distribution and homogeneity of variance using appropriate tests (homogeneity of variance was considered present when *P* > 0.1). Paired or unpaired *t*-tests were employed for continuous variables, with nonparametric tests used for data not assuming a Gaussian distribution. Chi-square or Fisher exact tests were utilized to compare distribution differences between two groups. Spearman correlation assessed variable correlations. Time-to-event outcomes were evaluated using Kaplan–Meier curves with log-rank tests. The determination of sample size was based on statistical power analysis to ensure sufficient ability to detect a pre-specified effect size. We assumed a moderate effect size, with an alpha level set at 0.05, and a power (1-β) set at 0.80. A significance level of *P* < 0.05 (two-tailed) was considered statistically significant.

The remaining materials and methods can be found in the supplementary materials.

## Results

### MAGI3 is identified as a novel prognostic indicator in ccRCC and is involved in MAPK signaling

Through differential gene expression analysis across multiple ccRCC datasets, we identified 984 differentially expressed genes (384 upregulated and 600 downregulated), with pathway enrichment highlighting significant involvement in cancer-related signaling pathways (Supplementary Fig. 1A). Notably, six of the top 20 enriched pathways were associated with the MAPK cascade (Supplementary Fig. 1B), underscoring its significance in ccRCC development. Within the subset of MAPK-related genes, 21 genes, including MAGI3, exhibited a notable correlation with the overall survival of ccRCC patients (Supplementary Fig. 1C). Despite MAGI3 not having been previously associated with ccRCC, its role in regulating ERK activation, as reported in our earlier publications [34–37], prompted a focused exploration into how MAGI3 expression influences ccRCC initiation and progression through ERK signaling.

**Fig. 1:**
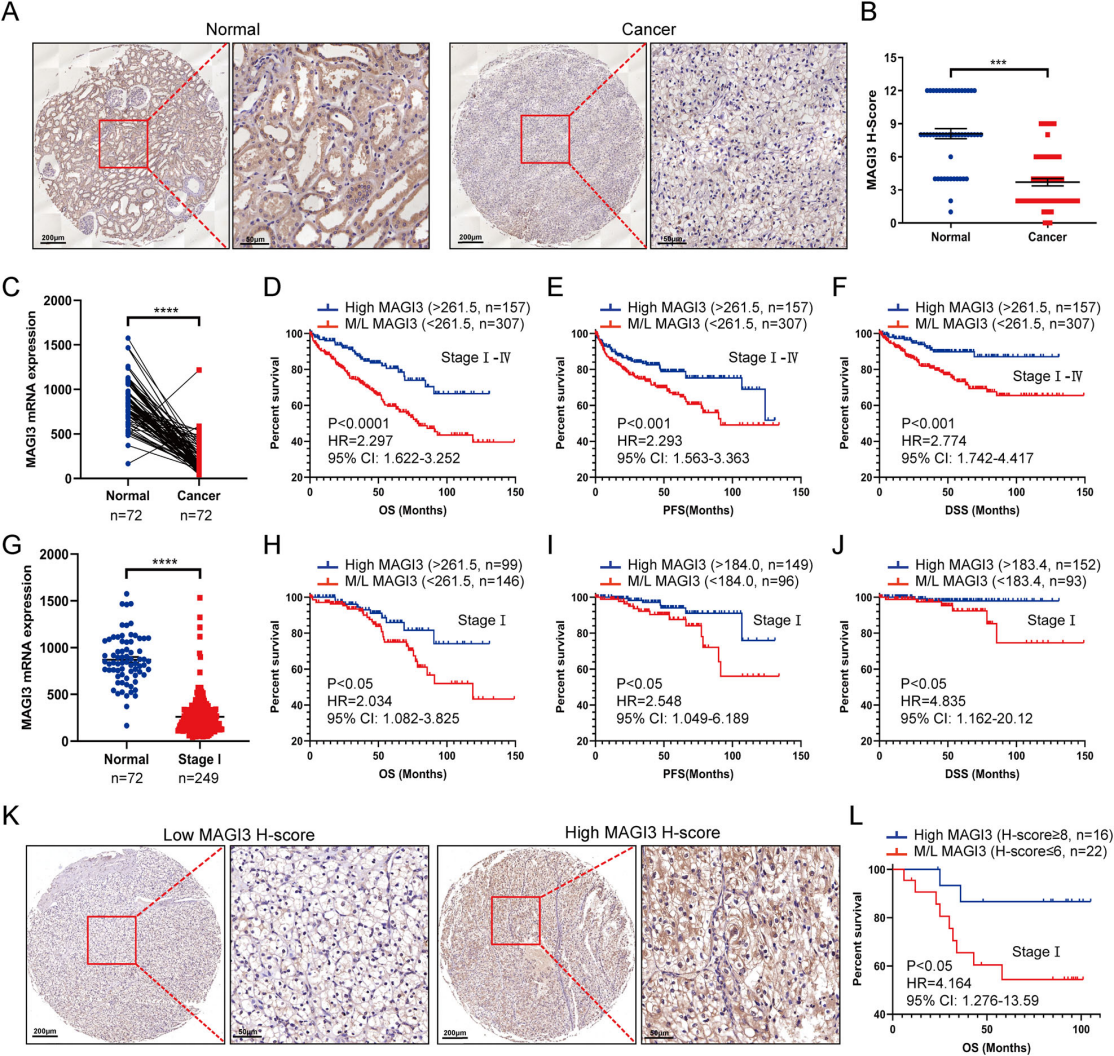
Dysregulated low levels of MAGI3 correlate with poor prognosis in ccRCC. **A**, **B** Immunohistochemical (IHC) analysis of MAGI3 protein in ccRCC specimens. Representative images of MAGI3 staining in adjacent tissues or tumors (**A**). Scale bars: 200 μm. Magnified images of dashed areas are displayed in the right panels. Scale bars: 50 μm. Dot plot showing the corresponding quantification of the MAGI3 H-score (**B**). Data presented as mean ± SEM. Statistical significance was calculated by the Mann–Whitney test. ***P < 0.001. **C** Dot plot comparing the mRNA expression level of MAGI3 in TCGA ccRCC specimens with paired normal tissues. Statistical significance is determined using the paired *t*-test (****P < 0.0001). **D**–**F** TCGA ccRCC patients were stratified into two groups based on ccRCC specimens MAGI3 expression cutoff at 261.5 (reads per kilobase of transcript, per million mapped reads, RPKM): high and middle-to-low (M/L). Kaplan–Meier survival plots for overall survival (OS) (**D**), progression-free survival (PFS) (**E**) and disease-specific survival (DSS) (**F**) of the two groups (log-rank test). **G** Dot plot showing the mRNA expression level of MAGI3 in tumor tissues and adjacent tissues of TCGA ccRCC patients with stage I. Statistical significance was calculated using the unpaired *t*-tests (****P < 0.0001). Kaplan–Meier survival plots for OS (**H**), PFS (**I**) and DSS (**J**) according to MAGI3 mRNA level in tumor tissue of TCGA ccRCC patients with stage I (log-rank test). **K**, **L** Prognostic analysis of OS according to MAGI3 histochemistry score (H-score) in tumor tissue of ccRCC patients with stage I from Chinese cohort. Representative images with different MAGI3 H-scores (**K**). Scale bars: 200 μm. The magnified images are displayed in the right panels. Scale bars: 50 μm. Kaplan–Meier survival plots for OS of patients with MAGI3 at high levels (H-score ≥ 8) and MAGI3 at middle-to-low levels (M/L, H-score ≤ 6) (log-rank test) (**L**).

To validate MAGI3 downregulation in ccRCC, we examined protein levels in 110 ccRCC samples from a Chinese cohort, revealing a substantial decrease compared to normal renal tissues (Fig. 1A, B). mRNA analysis also showed significantly reduced MAGI3 levels in ccRCC tissues, consistent with TCGA data (Fig. 1C) and GEO datasets (GSE16449, GSE53757, and GSE66271) (Supplementary Fig. 1D–F).

To assess MAGI3’s clinical relevance, the TCGA database excluded patients undergoing adjuvant therapy. The remaining cohort was divided into high (H, >261.5 RPKM) and middle-to-low (M/L, <261.5 RPKM) MAGI3 expression groups. Kaplan–Meier analysis revealed a substantial divergence, particularly within the M/L group, associated with lower 10-year overall survival (OS) (32.7% vs. 58.5%), progression-free survival (PFS) (37.9% vs. 48.3%), and disease-specific survival (DSS) (51.7% vs. 78.6%) rates (Fig. 1D–F, Supplementary Table 1). Notably, in stage I ccRCC patients, M/L MAGI3 expression (<261.5 RPKM) correlated with markedly reduced 10-year OS (45.5% vs. 74.6%), PFS (55% vs. 69.8%), and DSS (77.9% vs. 98%) compared to high MAGI3 expression (Fig. 1G–J, Supplementary Table 2). Consistent findings were validated across independent ccRCC cohorts, confirming the correlation between M/L MAGI3 levels [Histochemistry score (H-score) ≤6)] and an unfavorable prognosis, particularly at the protein level in stage I cases (Fig. 1K, L). Cox univariate and multivariate analysis identified MAGI3 protein level as an independent prognostic factor for stage I ccRCC patients (Supplementary Table 3). These results establish a significant association between reduced MAGI3 levels (M/L) and an unfavorable prognosis in ccRCC patients, revealing MAGI3 as a promising novel prognostic marker and providing insights into its pivotal role in ccRCC initiation and progression.

### Interaction between MAGI3 and MAS receptor

**Fig. 2:**
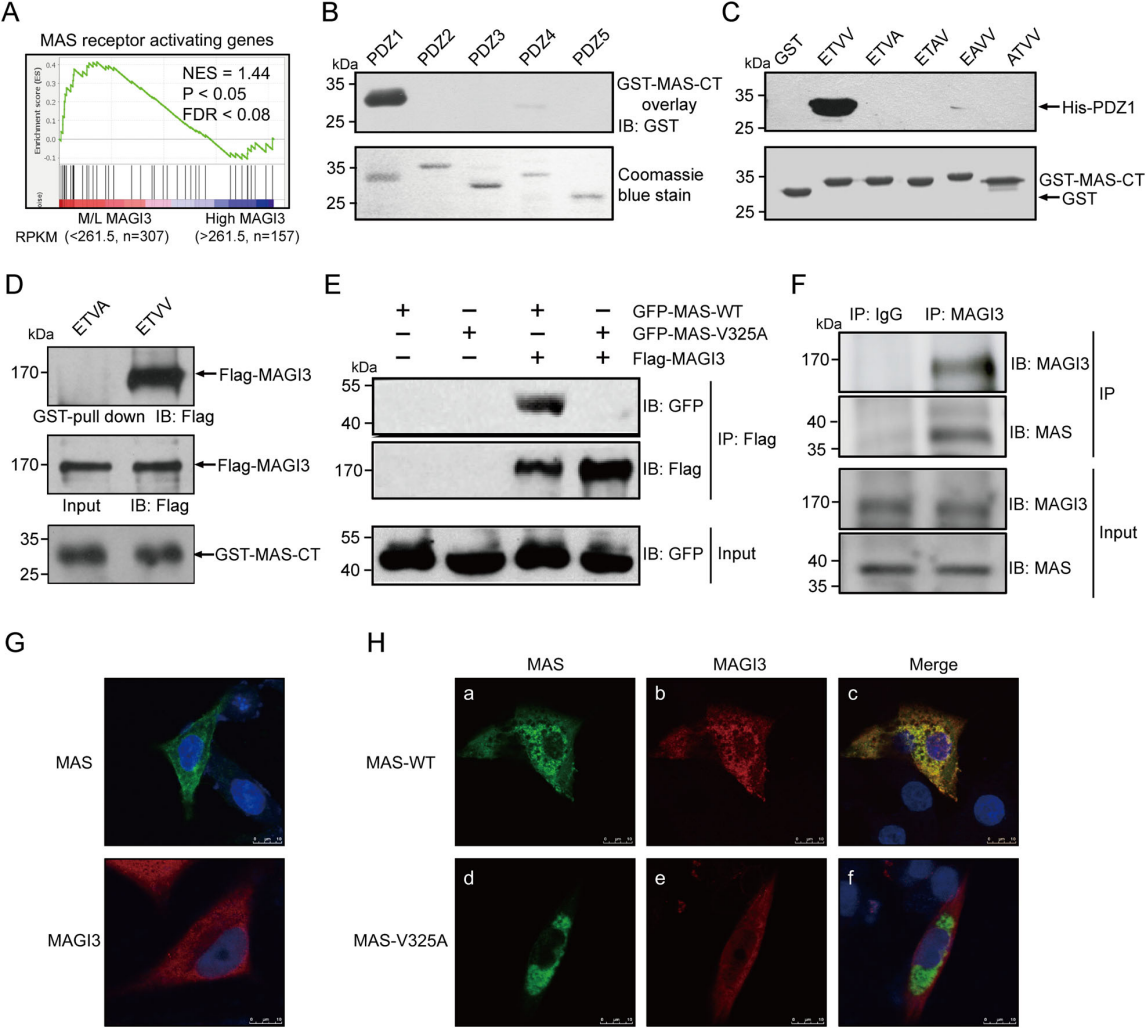
MAGI3 specifically interacts with MAS through its carboxyl terminus. **A** GSEA enrichment plots showing significant activation of MAS receptor in ccRCC specimens with low MAGI3 expression from the TCGA dataset. **B** The carboxyl terminus of MAS (MAS-CT) specifically binds to MAGI3 PDZ1 domains in an overlay assay. PDZ proteins (His tag) are separated through SDS-PAGE, and the isolated proteins are overlaid with GST-MAS followed by Western blot analysis. **C** The binding between MAS-CT and MAGI3 PDZ1 is disrupted when the ETVV motif in MAS-CT is mutated. GST fusion proteins of wild-type and mutant MAS-CT were employed for pull-down assays, and immunoblotting utilized an anti-His antibody. **D** The interaction between MAS-CT and MAGI3 is dependent on the ETVV motif at the C-terminal. In the pull-down assay, COS-7 cells were transfected with Flag-tagged MAGI3, the cell lysates were pulldown with either wild-type GST-MAS-CT or the ETVA mutant, followed by Western blot analysis. **E** Co-immunoprecipitation assay demonstrates the interaction between full-length MAS and MAGI3 through the ETVV motif at the MAS C-terminal. COS-7 cells were transfected with GFP-MAS WT or GFP-MAS V325A, both in the presence or absence of co-transfection with Flag-MAGI3, followed by Western blot analysis. **F** Co-immunoprecipitation of endogenous MAGI3 and MAS proteins in mouse kidney tissue. **G**, **H** Immunofluorescence co-localization of MAS and MAGI-3. BHK cells were transfected with GFP-MAS WT or GFP-MAS V325A, both in the presence or absence of co-transfection with Flag-MAGI3, and the co-localization was visualized through merging of individual images, Scale bars: 10 μm. Impaired colocalization was observed when the MAS V325A mutation was present.

MAGI3, recognized for its scaffolding role with five PDZ domains, forms multi-protein complexes. To gain a deeper understanding of MAGI3’s role in ccRCC, it is essential to identify its binding partners within renal cells. A previous proteomic analysis suggested a potential interaction between MAGI3 and the MAS receptor in human kidney cells, although without verification [38]. The potential interaction with the MAS receptor in renal cells was identified through GSEA analysis of the MAGI3 M/L group in ccRCC, indicating MAGI3’s involvement in MAS activation (Fig. 2A). Our previous investigation revealed a PDZ-binding motif at the carboxyl terminus (CT) of MAS [34], implying a direct association between MAGI3 and MAS signaling in ccRCC.

Through overlay assays, we confirmed a robust interaction between MAS’s CT and MAGI3’s first PDZ domain (PDZ1) (Fig. 2B). Furthermore, by generating mutants in MAS-CT’s PDZ-binding motif (PBM) and conducting GST pull-down assays, we determined that mutations in any PBM residue abolished the interaction with MAGI3 PDZ1 (Fig. 2C). This specific binding of MAS-CT to full-length MAGI3 was confirmed by GST pull-down assays (Fig. 2D). Co-transfection of Flag-MAGI3 and wild-type GFP-MAS in cellular experiments confirmed their interaction via co-immunoprecipitation assays; however, this interaction was disrupted by the MAS point mutant V325A (Fig. 2E), consistent with prior findings (Fig. 2C, D). Importantly, the presence of the endogenous MAS/MAGI3 complex was detected in mouse kidney tissue (Fig. 2F).

Immunofluorescence co-localization studies provided additional evidence of the MAGI3 and MAS interaction. Co-transfection of wild-type MAS and MAGI3 showed substantial co-localization (Fig. 2H a-c), while the MAS V325A mutant exhibited minimal co-localization with MAGI3 (Fig. 2H d-f).

Collectively, these findings provide evidence of a physical association between MAS and MAGI3 in renal cells, facilitated by the PDZ1 domain of MAGI3 and the C-terminal of MAS.

### MAGI3 suppresses MAS-mediated cell proliferation in ccRCC

To elucidate the biological significance of MAGI3, we analyzed the TCGA ccRCC dataset, stratifying data into MAGI3 expression high and middle/low groups. GSEA revealed significant enrichment of the cell proliferation gene signature in the MAGI3 M/L group (Supplementary Fig. 2A). Reduced MAGI3 expression showed a strong association with larger tumor size (Supplementary Fig. 2B), revealing the link between MAGI3 and cell proliferation in ccRCC. Considering that cell proliferation is a critical hallmark often driven by MAPK/ERK activation, and the MAS receptor is associated with promoting cell growth [39–41], we explored the impact of the MAS/MAGI3 axis on ccRCC cell proliferation.

**Fig. 3:**
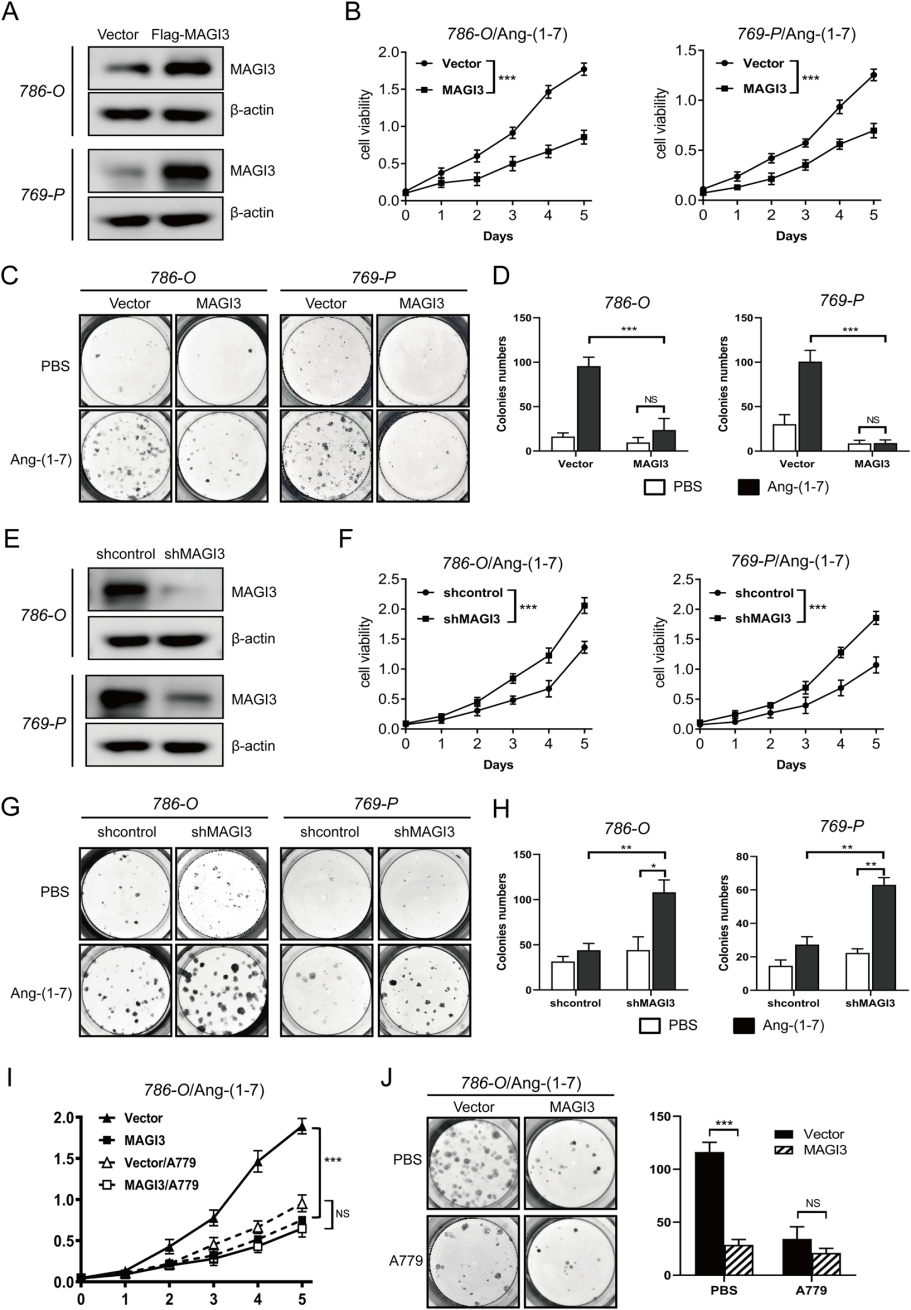
MAGI3 inhibits proliferation of ccRCC cells. **A** MAGI3 overexpression was confirmed via Western blot analysis in 786-O and 769-P cells following transfection with lentivirus carrying MAGI3 coding sequence or control vectors. **B** Increased MAGI3 expression reduced cell viability in 786-O and 769-P cells, as assessed over a 5-day period using a CCK8 assay. **C**, **D** Upregulated MAGI3 expression decreased colony formation ability, evident in crystal violet staining and photographs of ccRCC cell colonies formed over a 10-day culture period (**C**). Quantitative analysis shows a reduced number of colonies (**D**). **E** MAGI3 knockdown was confirmed in 786-O and 769-P cells using lentivirus-mediated delivery of shRNA, as evidenced by Western blot analysis. **F** Decreased MAGI3 expression enhanced cell viability, observed over a 5-day period through CCK8 assays in 786-O and 769-P cells. **G**, **H** Reduced MAGI3 expression increased colony formation in ccRCC cells, as observed through crystal violet staining and colony photography (**G**). Quantitative analysis indicated an increased number of colonies (**H**). **I**, **J** The inhibitory effect of MAGI3 on cell proliferation was abolished by blocking the MAS receptor with A779, as demonstrated in CCK8 (**I**) and cell colony formation (**J**) assays in MAGI3-overexpressing 786-O cells and control cells treated with A779. Statistical significance is indicated by ***(*P* < 0.001); NS non-significant.

Stable transfections of 786-O and 769-P ccRCC cells were established with control or MAGI3 constructs (Fig. 3A). Cell growth, assessed through CCK8 colorimetric and colony-formation assays under low serum conditions in the presence of Ang-(1-7), revealed that MAGI3 overexpression significantly reduced Ang-(1-7)-induced cell proliferation (Fig. 3B) and colony formation (Fig. 3C, D) in ccRCC cells. Conversely, depleting MAGI3 expression in 786-O and 769-P ccRCC cells (Fig. 3E) significantly increased cell proliferation (Fig. 3F) and clonogenicity (Fig. 3G, H). Additionally, MAGI3 overexpression induced cell cycle arrest in the G0/G1 phase in ccRCC cells (Supplementary Fig. 2C).

Importantly, the inhibitory impact of MAGI3 on ccRCC cell proliferation (Fig. 3I) and colony formation (Fig. 3J) was nullified with the administration of the MAS receptor antagonist A779, indicating that MAGI3 constrains cell proliferation via the MAS receptor.

### MAGI3 suppresses MAS-mediated ERK signaling pathway in ccRCC cell

The MAPK/ERK pathway, a critical regulator of cellular processes like proliferation, has been identified to associate with MAGI3 (Supplementary Fig. 1B). GSEA analysis further reveals a significant enrichment of the gene signature related to ERK activation in the MAGI3 M/L group of ccRCC, establishing an association between MAGI3 and the activation of MAPK/ERK signaling in ccRCC cells (Supplementary Fig. 3A).

**Fig. 4:**
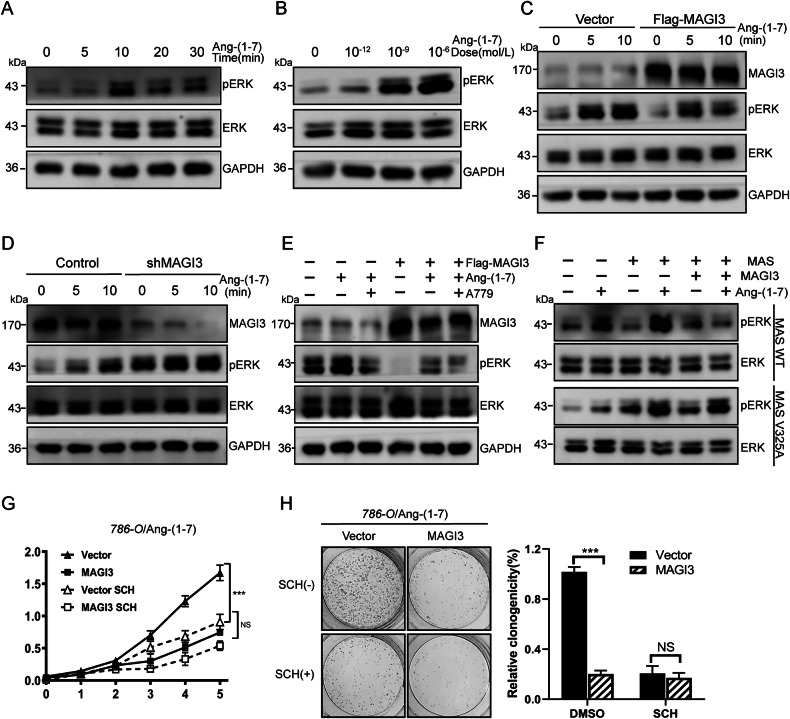
MAGI3 inhibits MAS-mediated ERK activation. **A** Time-dependent analysis of ERK activation in 786-O cells stimulated with 10 μM Ang-(1-7). Phosphorylated ERK1/2 was assessed at different time points following Ang-(1-7) treatment in serum-starved 786-O cells. **B** Dose-response evaluation of Ang-(1-7)-induced ERK activation in 786-O cells. Various concentrations of Ang-(1-7) were administered to serum-starved COS-7 cells for 5 minutes, and ERK phosphorylation was measured. **C** MAGI3 inhibits Ang-(1-7)-induced ERK activation in 786-O cells. 786-O cells, transfected with Flag or Flag-MAGI3 underwent serum starvation and were stimulated with 10 μM Ang-(1-7) for varying durations. MAGI3 exhibited substantial inhibition of ERK phosphorylation, with the most pronounced effect observed at 10 minutes. **D** MAGI3 knockdown promotes MAS-mediated ERK activation in 786-O cells. 786-O cells with reduced MAGI3 expression and control cells were serum-starved and stimulated with 10 μM Ang-(1-7) for 5 min. ERK phosphorylation was measured via Western blot. **E** Specific inhibition of Ang-(1-7)-induced ERK activation in 786-O cells by blocking MAS activation with A779. Serum-starved 786-O cells transiently overexpressing Flag or Flag-MAGI3 were treated with the MAS antagonist A779 (1 μM) and 10 μM Ang-(1-7) for 5 min. Both MAGI3 overexpression and A779 treatment effectively suppressed MAS-mediated ERK phosphorylation. **F** MAGI3’s interaction with MAS contributes to the inhibition of MAS-induced ERK activation. 786-O cells, transfected with MAS or MAS V325A mutant, and MAGI3 were serum-starved and stimulated with 10 μM Ang-(1-7) for 5 min. Western blot analysis revealed that MAGI3 significantly inhibited MAS-mediated ERK activation but had little effect on MAS V325A. Ang-(1-7)-induced ERK activation in the absence of MAGI3 was used as a control. **G**, **H** MAGI3’s inhibitory effect on cell proliferation is abolished when the ERK pathway is blocked with SCH772984 in MAGI3-overexpressing 786-O cells, as demonstrated by CCK8 (**G**) and cell colony formation assays (**H**). Statistical significance is denoted by ***(*P* < 0.001); NS indicates non-significance.

To explore the connection between the MAPK/ERK pathway and Ang-(1-7)/MAS signaling, 786-O cells transfected with the MAS receptor were stimulated with varying Ang-(1-7) concentrations for different durations. ERK phosphorylation peaked at 10 minutes (Fig. 4A and Supplementary Fig. 3B), exhibiting a concentration-dependent increase, with maximum activation observed at an Ang-(1-7) concentration of 10^-6 ^M (Fig. 4B and Supplementary Fig. 3C).

In investigating MAGI3’s molecular mechanism, we examined Ang-(1-7)-induced ERK activation in 786-O cells with MAGI3 overexpression and knockdown. MAGI3 overexpression significantly reduced Ang-(1-7)-induced ERK phosphorylation (Fig. 4C), while MAGI3 depletion amplified ERK activity (Fig. 4D). A779, a MAS receptor antagonist, almost completely inhibited Ang-(1-7)-induced ERK activation (Fig. 4E). These findings suggest that Ang-(1-7) primarily signals through MAS to activate the ERK pathway, and MAGI3 can restrain this activation. The inhibitory effect of MAGI3 on ERK activation is dependent on the MAS/MAGI3 interaction, as demonstrated by reduced phosphorylated ERK levels in cells with wild-type MAS but not in those with the MAS-V325A mutant, disrupting the MAS/MAGI3 interaction (Fig. 4F and Supplementary Fig. 3D).

Examining the impact of blocking the ERK signaling pathway on MAGI3’s effects on MAS-mediated cell functions, we found that the ERK inhibitor SCH772984 effectively reduced Ang-(1-7)-induced proliferation and colony formation in 786-O cells, confirming the crucial role of the ERK signaling pathway in MAS-mediated cell growth. Notably, SCH treatment showed no additional inhibition of cell growth in MAGI3-overexpressing cells, indicating that MAGI3 hampers MAS-mediated renal cell proliferation by suppressing the ERK signaling pathway (Fig. 4G, H). These results highlight the significant contribution of the MAS/MAGI3/ERK axis to ccRCC cell proliferation.

**Fig. 5:**
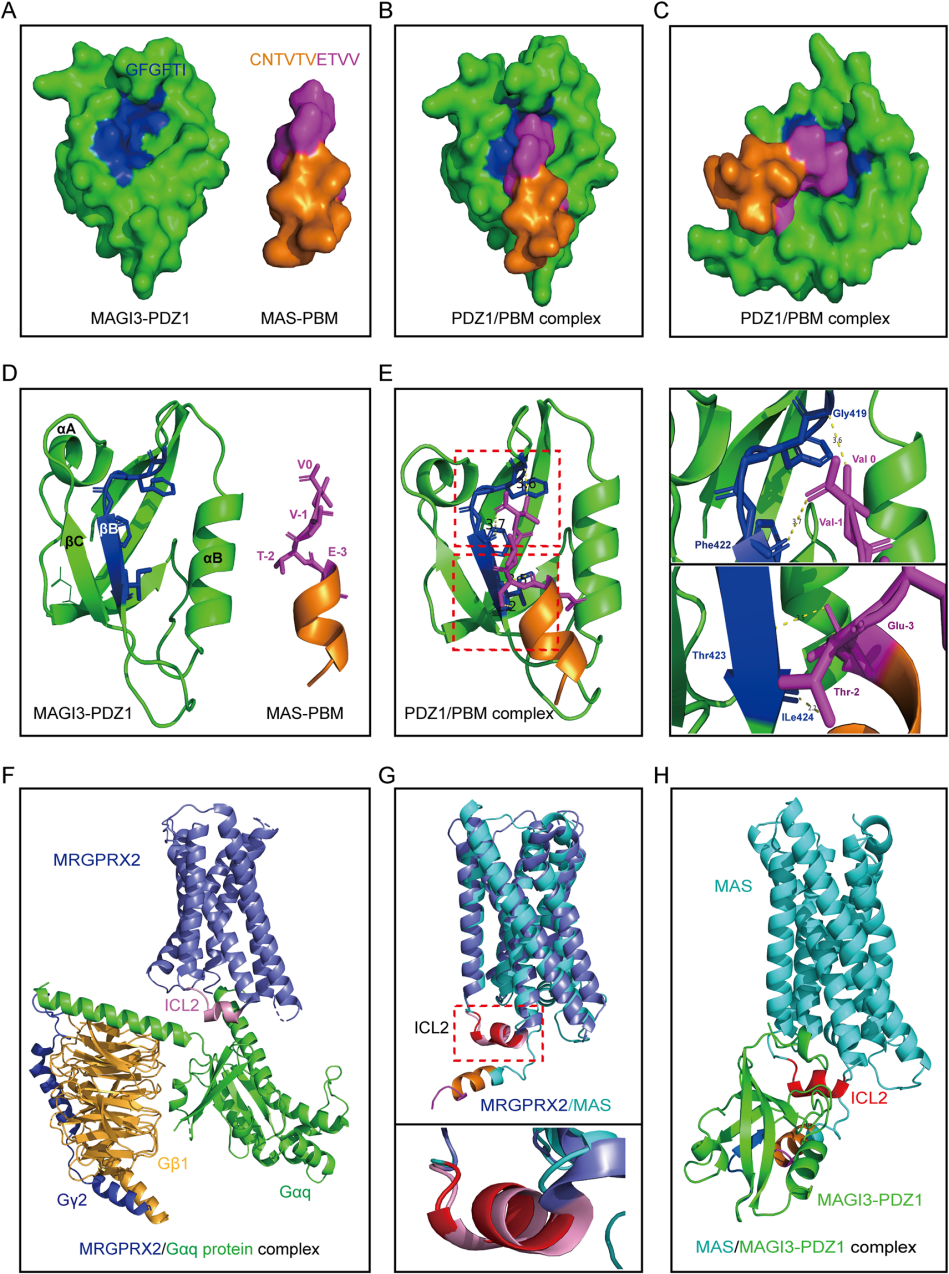
Molecular dynamics simulation of the carboxyl terminus of MAS bound with MAGI3 to block the coupling of G protein with the intracellular loop 2 (ICL2) of MAS. **A** Predicted AlphaFold models of MAGI3-PDZ1, with the carboxylate binding loop (Gly-419, Phe-420, Gly-421, Phe-422, Thr-423, Ile-424) highlighted in blue, and other residues in green. MAS-PBM is colored in oranges (Cys-316, Asn-317, Thr-318, Val-319, Thr-320, Val-321) and magentas (Glu-322, Thr-323, Val-324, Val-325). **B**, **C** Molecular simulation of MAGI3-PDZ1/MAS-PBM complex. Front (**B**) and side (**C**) views of the interaction between MAGI3-PDZ1/MAS-PBM. **D**. Ribbon diagram representation of MAGI3-PDZ1 and MAS-PBM structure. Colors correspond to those in Fig. 5A. The “ETVV” motif of MAS is labeled. **E** Stereoview of the detailed interface of MAGI3-PDZ1/MAS-PBM complex. Dotted lines represent chemical bonds. Right panels show magnifications of the wireframe areas on the left. The “GFGFTI” motif of MAGI3-PDZ1 and “ETVV” motif of MAS are labeled. **F** Crystal structure of MRGPRX2-Gq signaling complex (PDB: 7S8N). The ICL2 that interacts with Gαq protein on MRGPRX2 is colored in pink. **G** Structural comparison of MAS AlphaFold model with MRGPRX2. Lower panel show magnifications of the wireframe area on the upper. **H** Molecular dynamics simulation of MAS/MAGI3-PDZ1 complex, indicating the interaction of MAGI3-PDZ1 with MAS-PBM obscuring the ICL2 on MAS that interacts with G protein.

To delve into the mechanisms of MAGI3 disrupting the ERK pathway via interaction with MAS, we utilized AlphaFold for structural prediction. Using protein files from the PDB database, we discerned detailed protein surfaces. Structural analysis highlighted a hydrophobic pocket in MAGI3-PDZ1 and an alignment of MAS receptor’s C-terminal with the class I PDZ-binding motif (PBM), forming the basis for their reciprocal binding (Fig. 5A–C).

Further analysis revealed the chemical bonds between MAS’s C-terminal motif “ETVV” and the hydrophobic loop sequence “GFGFTI” of MAGI3’s PDZ1, emphasizing the crucial role of the “ETVV” motif in MAGI3-MAS binding (Fig. 5D, E, Supplementary Table 4), consistent with our previous overlay and GST pulldown experiments (Fig. 2B, C). Subsequently, we investigated the impact of MAGI3-PDZ1 and MAS interaction on MAS’s coupling with G-proteins. Literature and structural studies suggested the intracellular loop 2 (ICL2) as the potential G-protein coupling site for MAS, similar to MRGPR2 (Fig. 5F, G, Supplementary Table 5) [42–44]. Notably, the interaction orchestrated by MAS’s “ETVV” motif with MAGI3-PDZ1 significantly obstructed MAS’s ICL2 coupling with G-proteins (Fig. 5H). These findings lead us to propose that MAGI3’s interaction with MAS impedes G-protein coupling with the MAS receptor, ultimately inhibiting the activation of the ERK pathway.

### MAGI3 regulates cell proliferation through the MAS/ERK axis in vivo

**Fig. 6:**
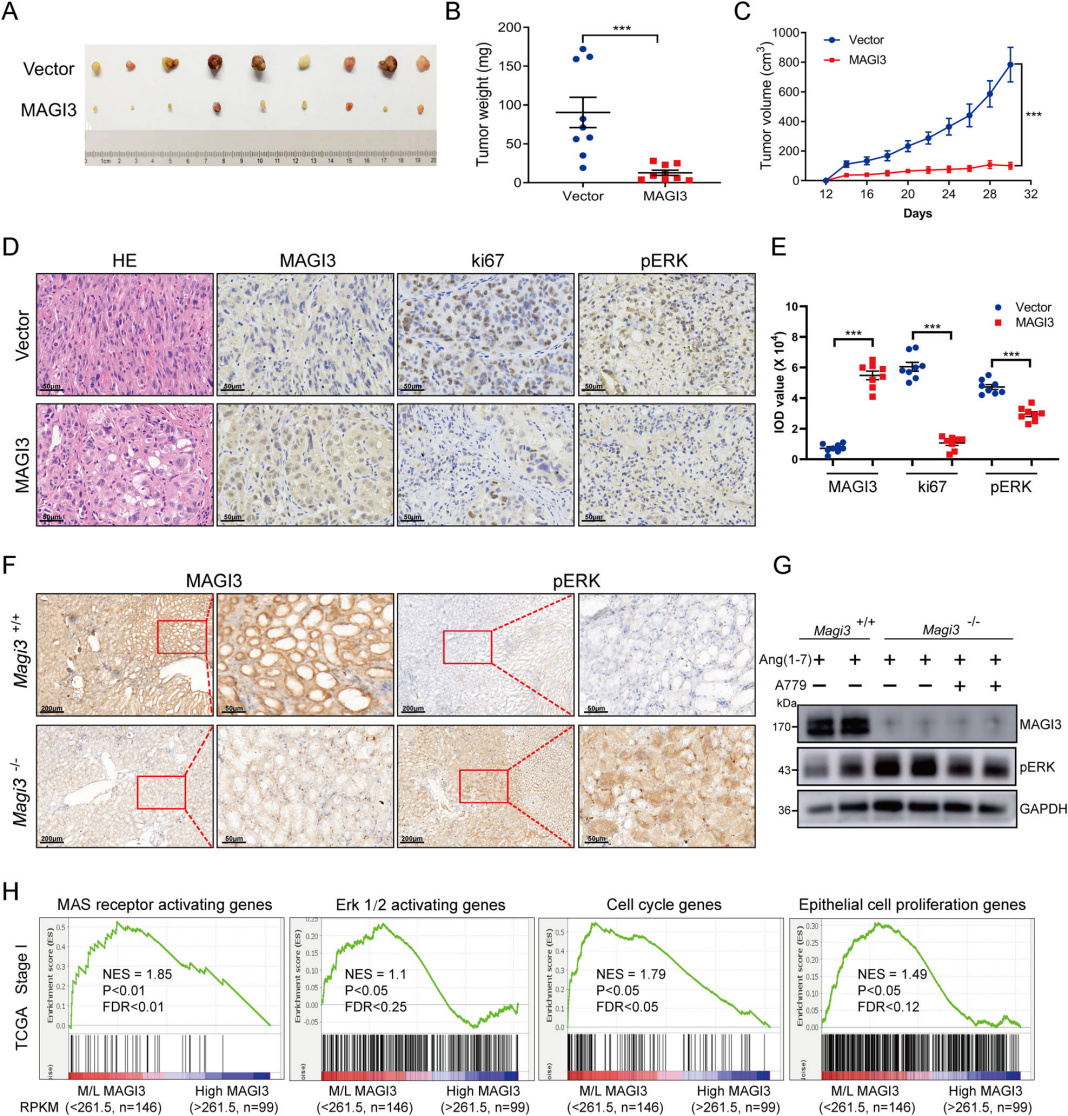
MAGI3 inhibits MAS-induced ccRCC tumor proliferation in vivo. **A**–**C** Upregulation of MAGI3 inhibits MAS-induced tumor growth in xenografted ccRCC cells in BLAB/c nude mice. After subcutaneously implanting 786-O cells overexpressing MAGI3 or control vector cells, subcutaneous injections of 0.5 mg/kg Ang-(1-7) were administered every 24 h upon tumor formation. Tumor volumes were monitored at specified intervals, with the largest tumors allowed to reach 1000 mm^3^. Upon sacrifice, tumor weights were recorded. Displayed are representative images of xenograft tumors (**A**) tumor weight (**B**) and tumor growth curves (**C**). **D**, **E** Downregulation of pERK and Ki-67 levels in transplanted tumors with MAGI3 overexpression. Paraffin sections from the 786-O ccRCC tumors in nude mice were subjected to immunohistochemistry staining, utilizing MAGI3, pERK, and Ki-67 antibodies. Scale bars: 50 μm. Data are presented as mean ± SEM. Significance levels are indicated as *, **, *** (*P* < 0.05, *P* < 0.01, *P* < 0.001). **F** Representative immunohistochemistry staining of MAGI3 and pERK in kidney tissues from *Magi3*^−/−^ or *Magi3*^+/+^ mice. Scale bars: 200 μm. Right panels exhibit magnifications of the dashed areas on the left. Scale bars: 50 μm. **G** *Magi3* knockdown fails to trigger ERK phosphorylation by blocking MAS activation with A779. Western blot analysis of pERK in kidney tissues from *Magi3*^−/−^ or *Magi3*^+/+^ mice, with or without A779 pretreatment. **H** MAGI3’s impact on ccRCC cell proliferation mediated by the MAS/ERK axis. Enrichment plots from gene set enrichment analysis (GSEA) reveal significant activation of MAS, ERK, cell cycle, and proliferation pathways in ccRCC specimens with middle-low (M/L, <261.5 RPKM) MAGI3 expression from the TCGA ccRCC patients with stage I.

In tumor-bearing experiments with BLAB/c nude mice injected with MAGI3-overexpressing 786-O cells and treated with Ang-(1-7), MAGI3 overexpression significantly inhibited xenograft tumor growth. These tumors exhibited reduced weight and volume, along with decreased expression of Ki67 and phosphorylated ERK, emphasizing MAGI3’s regulatory role (Fig. 6A–E).

To confirm MAGI3’s impact on ERK activation in renal cell in vivo, *Magi3*-knockout (KO) mice were generated. Immunohistochemical results consistently showed a significant accumulation of phosphorylated ERK in the kidneys of *Magi3*-deficient mice (Fig. 6F). Importantly, Western blot analysis demonstrated that *Magi3* knockdown did not induce ERK pathway activation in mice treated with A779 (Fig. 6G).

The in vivo mouse experiments, along with clinical data analysis (Supplementary Figs. 2A, 3A, and Fig. 2A), unequivocally demonstrate a substantial rise in MAS-mediated ERK pathway activation and its pro-proliferative effects in the absence of MAGI3, even in the early stages of ccRCC (Fig. 6H). In summary, these findings collectively confirm the pivotal role of MAGI3 in inhibiting renal cell proliferation by modulating the MAS/ERK axis in both in vitro and in vivo settings.

### MAGI3 modulates Sunitinib sensitivity in ccRCC cells

**Fig. 7:**
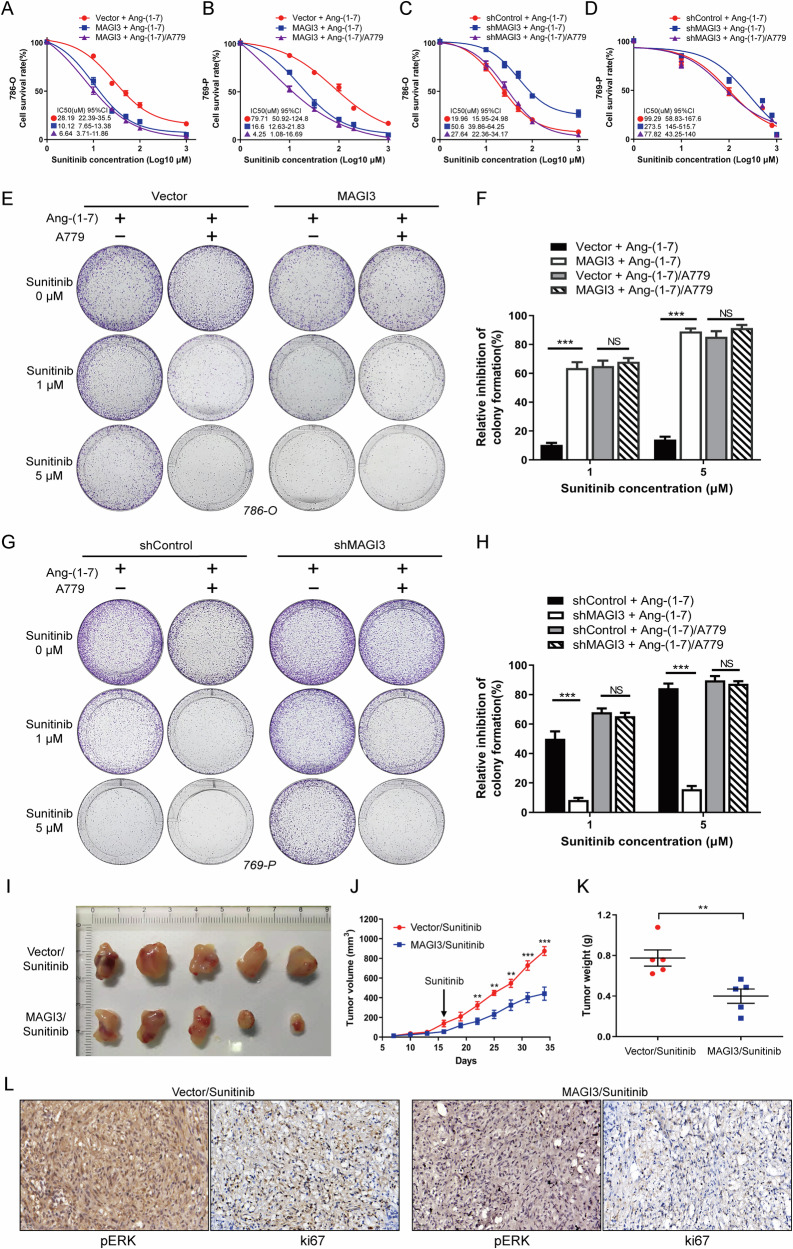
MAGI3 improves Sunitinib sensitivity by suppressing the MAS/ERK signaling pathway. **A**, **B** MAGI3 overexpression reduces Sunitinib IC50 in 786-O and 769-P cells with MAS activation. Survival curves of MAGI3-overexpressing 786-O (**A**) and 769-P (**B**) cells treated with increasing sunitinib concentrations for 48 h, with or without A779 pretreatment. Mean ± SEM; *n* = 3; two-way ANOVA. **C**, **D** With MAS activation, MAGI3 knockdown increases sunitinib IC50 in 786-O and 769-P cells. Survival curves of MAGI3 knockdown 786-O (**C**) and 769-P (**D**) cells treated with increasing sunitinib concentrations for 48 h, with or without A779 pretreatment. Mean ± SEM; *n* = 3; two-way ANOVA. **E**, **F** MAGI3 overexpression significantly inhibits colony formation in sunitinib-treated 786-O cells with MAS activation. Quantification analysis of colony formation assays in MAGI3-overexpressing 786-O cells treated with increasing concentrations of sunitinib for 10 days, with or without A779 pretreatment. Mean ± SEM; *n* = 3; one-way ANOVA. **G**, **H** MAGI3 knockdown significantly promotes colony formation in sunitinib-treated 769-P cells in the presence of MAS activation. Quantification analysis of colony formation assays in MAGI3 knockdown 769-P cells treated with increasing concentrations of sunitinib for 10 days, with or without A779 pretreatment. Mean ± SEM; *n* = 3; one-way ANOVA. **I**–**L** MAGI3 enhances Sunitinib sensitivity in renal cell carcinoma xenografts. Nude mice transplanted with MAGI3-overexpressing 786-O cells or control cells were treated with Ang-(1-7) and Sunitinib. Representative images of xenograft tumors (**I**) tumor growth curves, two-way ANOVA (**J**) and tumor weight (**K**) are shown. Representative IHC staining of pERK and ki67 in xenografted tumors are shown in **L**. Data are presented as mean ± SEM. *P < 0.05, **P < 0.01, ***P < 0.001.

Given that the inhibition of the ERK signaling pathway is a major mechanism through which Sunitinib suppresses tumor cell proliferation, we examined the impact of MAGI3 expression on Sunitinib sensitivity in ccRCC cells. Firstly, we analyzed the expression changes of MAIG3 in Sunitinib resistant renal cancer cells in the GSE64052 database, and the results showed that the mRNA level of MAGI3 was significantly downregulated in Sunitinib-resistant cells (Supplementary Fig. 4A). Subsequently, our cell experiments showed that overexpression of MAGI3 significantly reduced the IC50 of Sunitinib in 786-O cells (from 28.19 to 10.12 μM) and 769-P cells (from 79.71 to 16.60 μM) under Ang1-7 stimulation. Interestingly, MAGI3-induced cellular sensitivity to Sunitinib was not further enhanced with pre-treatment using A779 to block MAS activation (Fig. 7A, B). Conversely, depletion of MAGI3 expression increased the IC50 of Sunitinib in 786-O cells (from 19.96 to 50.60 μM) and 769-P cells (from 99.29 to 273.5 μM) under Ang1-7 stimulation. However, pre-treatment with A779 significantly mitigated the Sunitinib resistance induced by MAGI3 knockdown (Fig. 7C, D). Colony formation assays supported these findings, demonstrating that MAGI3 overexpression increased sensitivity in 786-O cells, while MAGI3 knockdown reduced sensitivity in 769-P cells. Importantly, pre-treatment with A779 nullified the changes in Sunitinib sensitivity resulting from MAGI3 manipulation (Fig. 7E–H).

In the Sunitinib-treated subcutaneous xenograft tumor model, MAGI3 overexpression led to significantly slowed tumor growth, reduced weight, and diminished levels of phosphorylated ERK and ki67, affirming the pivotal role of the MAS/MAGI3 axis in regulating Sunitinib sensitivity in ccRCC cells (Fig. 7I–L).

**Fig. 8:**
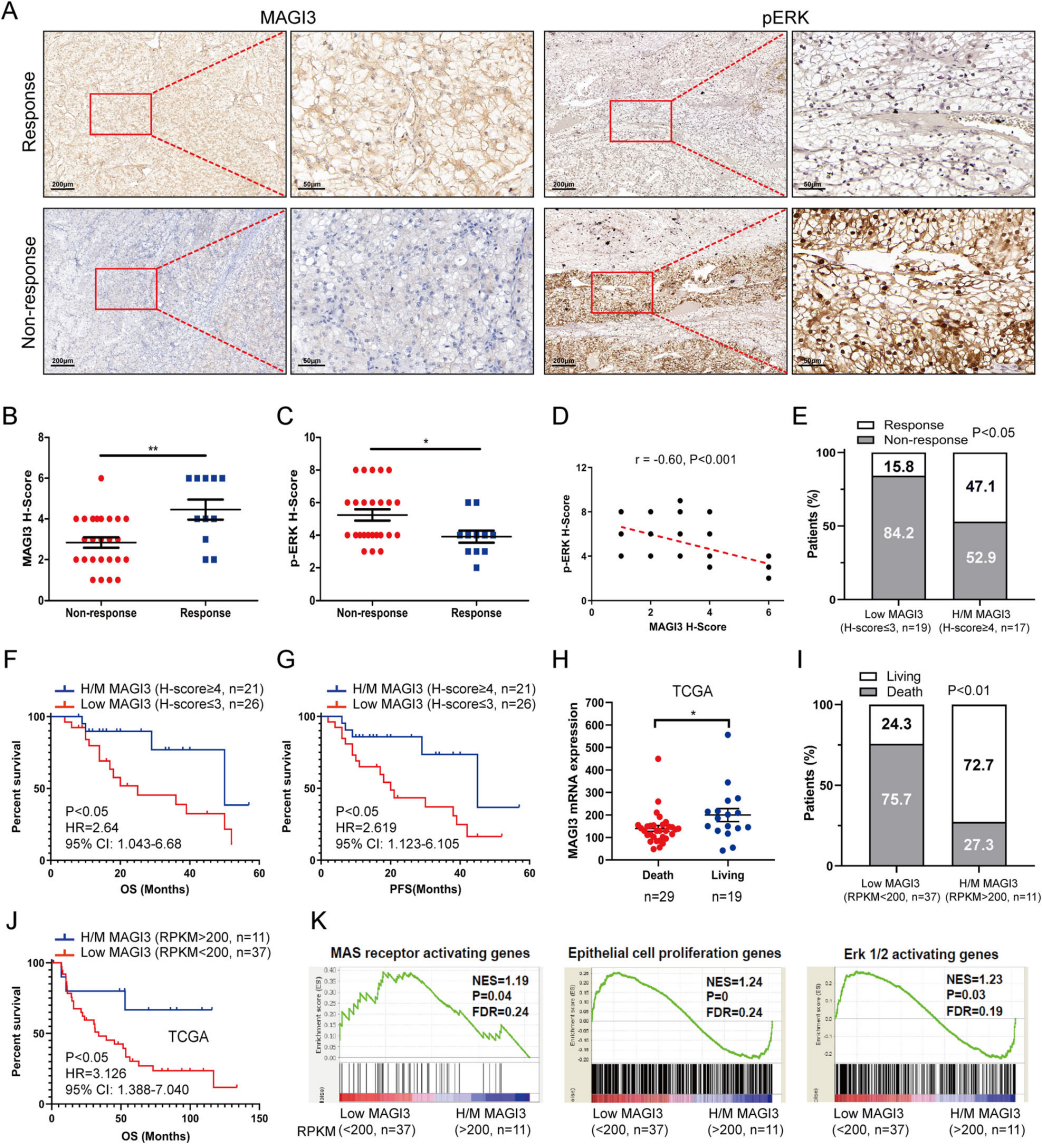
High MAIG3 levels associated with increased sensitivity to Sunitinib, and the reduction of ERK signaling in ccRCC specimens. **A** IHC analysis of MAGI3 and p-ERK in ccRCC specimens from 47 Sunitinib-treated patients. Representative images of MAGI3 and p-ERK staining in Sunitinib responders (R) and non-responders (NR). Scale bars: 200 μm. The right panels display magnified views of the dashed areas on the left. Scale bars: 50 μm. Quantification of MAGI3 H-score (**B**) and p-ERK H-score (**C**) in ccRCC specimens. Data is presented as mean ± SEM. Statistical significance was determined using the Mann–Whitney test. *P < 0.05, **P < 0.01. **D** Correlation analysis (Spearman) between MAGI3 H-score and p-ERK H-score in ccRCC specimens. **E** Evaluation of the response ratio to Sunitinib in patients with MAGI3 high-to-middle (H/M) levels (H-score ≥4) (chi-square test). Kaplan–Meier (KM) survival plots for overall survival (OS) (**F**) and progression-free survival (PFS) (**G**) based on MAGI3 H-score in ccRCC specimens (log-rank test). **H** Comparison of MAGI3 mRNA expression levels in ccRCC specimens between patients with favorable responses (Living) and those with poor responses (Death) to Sunitinib in the TCGA dataset of 48 patients. Data is presented as mean ± SEM, with statistical significance determined using the Mann–Whitney test. * indicates significance at *P* < 0.05. **I** ccRCC patients with MAGI3 mRNA levels above 200 RPKM (high-middle, H/M) had a notably higher response ratio to Sunitinib, as indicated by chi-square test results. **J** Kaplan–Meier survival plots demonstrate the association between MAGI3 mRNA expression levels and overall survival based on the log-rank test. **K** GSEA shows significant activation of MAS, proliferation, and ERK pathways in ccRCC specimens with low MAGI3 expression among patients treated with Sunitinib, derived from the TCGA dataset.

In a clinical study using IHC staining on ccRCC patients undergoing Sunitinib treatment, those with higher MAGI3 levels and lower phosphorylated ERK levels displayed a favorable response, while poor responders exhibited the opposite (Fig. 8A–C). A significant negative correlation between MAGI3 protein levels and phosphorylated ERK levels was identified (r = −0.6, Fig. 8D). Patients with MAGI3 in middle-to-high levels (H/M, H-score ≥4) demonstrated a substantially higher response rate to Sunitinib compared to those with lower levels (H-score ≤3) (47.1% vs. 15.8%, Fig. 8E), highlighting the association between MAGI3 expression and Sunitinib response in a clinical context. Kaplan–Meier survival analysis revealed that patients with lower MAGI3 H-scores had worse OS [Hazard Ratio (HR) = 2.64, 95% Confidence Interval (CI): 1.043–6.680] and PFS (HR = 2.619, 95% CI: 1.123–6.105) compared to those with MAGI3 H/M in H-scores (Fig. 8F, G).

This observation was validated by the TCGA dataset. Responders to Sunitinib treatment exhibited higher MAGI3 levels than non-responders (Fig. 8H). Patients with high-middle (H/M, MAGI3 mRNA levels >200 RPKM) levels showed a significantly higher response rate to Sunitinib treatment compared to those with low (L, MAGI3 levels <200 RPKM) levels (72.7% vs. 24.3%) (Fig. 8I). Kaplan–Meier analysis indicated that patients in the L group had worse OS compared to those in the H/M group, with an HR of 3.126 (95% CI: 1.388–7.04) (Fig. 8J, Supplementary Table 6). Additionally, the low-MAGI3 group exhibited notable enrichment of the gene signature associated with cell proliferation, MAS receptor activation, and ERK1/2 activation (Fig. 8K).

Taken together, these findings strongly suggest that the downregulation of MAGI3 expression in ccRCC patients initiates MAS/ERK signaling activation, contributing to Sunitinib resistance.

## Discussion

Surgical nephrectomy is a highly effective treatment for localized ccRCC patients. Traditional approaches generally do not incorporate additional adjuvant therapies, especially for low-risk or early-stage cases like stage I. However, due to the substantial heterogeneity of ccRCC, approximately 25% of localized patients face a risk of relapse post-surgery [45]. Thus, stratifying localized ccRCC is essential to optimize treatment outcomes.

Prognosis prediction in ccRCC has traditionally relied on tumor staging, histological grading, or lymphatic infiltration, offering insights into tumor anatomy and histology [46]. Yet, ccRCC’s remarkable heterogeneity leads to varying outcomes, even among patients with similar clinical characteristics [47]. This diversity arises from differences in molecular pathogenesis, including distinct gene and protein expressions, even within the same stage or grade [48–50]. Therefore, molecular markers, particularly specific gene expressions, hold the potential to significantly improve the precision of outcome predictions and enable more tailored treatment strategies for ccRCC patients [51].

This study establishes a significant correlation between reduced MAGI3 expression and an unfavorable prognosis in ccRCC, revealed through comprehensive analysis across multiple patient cohorts (Supplementary Fig. 1). In order to reflect the predictive effect of MAGI3 on the natural prognosis of ccRCC patients who did not receive postoperative adjuvant therapy, we removed patients who received adjuvant therapy after surgery from the TCGA database and conducted survival analysis on patients who only received surgical treatment. In addition, the ccRCC tissue we tested were also from patients who did not receive postoperative adjuvant therapy. The results show that reduced MAGI3 levels emerge as a substantial risk factor and prognostic indicator for adverse clinical outcomes, particularly in early-stage ccRCC. Among Stage I ccRCC patients, those with middle-to-low MAGI3 expression (<261.5 RPKM or H-score ≤ 6) experience a notable decrease in 10-year OS (45.5% vs. 74.6%), PFS (55% vs. 69.8%), and DSS (77.9% vs. 98%) compared to those with high MAGI3 expression (>261.5 RPKM or H-score ≥ 8) (Fig. 1). These findings position MAGI3 as a promising prognostic marker, enhancing outcome prediction accuracy and enabling effective patient stratification.

This study unveiled MAGI3’s anti-tumor role in ccRCC progression by inhibiting ERK activation induced by the Ang-(1-7)/MAS axis. Building upon our prior work, which linked the Ang-(1-7)/MAS axis to ccRCC cell migration [34], our current findings illustrate MAGI3’s ability to suppress cell proliferation mediated by Ang-(1-7)/MAS through ERK pathway inhibition in renal cells (Figs. 3 and 4, Supplementary Fig. 2). Further validation through subcutaneous xenograft models, *Magi3*-knockout mice, and clinical analyses underscored the crucial involvement of the MAGI3/MAS/ERK axis in RCC development (Fig. 6, Supplementary Figs. 2, 3). As the ERK pathway plays a pivotal role in cell survival, cycle progression, and proliferation [33], recent studies emphasizing Ang-(1-7)/MAS axis-induced ERK activation in ccRCC gain significance [52]. Our study revealed that MAGI3 significantly reduces Ang-(1-7)-induced ERK phosphorylation in a dose- and time-dependent manner through its interaction with the MAS receptor, shedding light on the critical role of the MAGI3/MAS/ERK axis in RCC development and progression.

Our study reveals a direct physical interaction between MAGI3 and the MAS receptor in renal cells, supported by multiple protein interaction assays and immunofluorescence co-localization studies (Fig. 2). This association is mediated through the PDZ1 domain of MAGI3 and the C-terminal of MAS. Structural analysis and dynamic simulation using AlphaFold suggest that MAS’s intracellular loop 2 (ICL2) serves as a potential G-protein coupling site (Fig. 5F, G). The interaction of MAS’s C-terminal motif “ETVV” and the hydrophobic groove of MAGI3-PDZ1, hinders MAS’s ICL2 from coupling with G proteins (Fig. 5D–H). This interference by MAGI3 inhibits the activation of the ERK pathway, providing insights into the modulation of MAS/ERK signaling.

This study highlights MAGI3’s substantial impact on modulating Sunitinib sensitivity in ccRCC, suggesting its potential as a predictor of Sunitinib response. MAGI3 overexpression enhances sensitivity, while knockdown reduces it in ccRCC (Fig. 7). Notably, manipulating MAGI3 doesn’t affect Sunitinib IC50 in ccRCC, post A779 treatment. Among patients receiving Sunitinib treatment, those with MAGI3 protein at H/M levels (H-score ≥4) show significantly improved survival (47.1% vs. 15.8%) (Fig. 8A–G), confirmed by independent datasets (Fig. 8H–J). GSEA indicates MAS/ERK signaling enrichment in ccRCC patients with low MAGI3 expression (Fig. 8K). These collective evidences emphasize the significant role of MAGI3 in regulating Sunitinib responsiveness in ccRCC cells through the MAS/ERK pathway.

As mentioned earlier, patients with MAGI3 levels in the middle to low range (H-score ≤ 6 or <261.5 RPKM) in stage I ccRCC demonstrated significantly lower survival rates compared to those with MAGI3-high levels (H-score ≥ 8 or >261.5 RPKM) (54.4% vs. 86.7% with a 5-year OS, Fig. 1K, L), suggesting the critical necessity for additional treatment interventions. Among patients with MAGI3 M/L levels, identified as a subset at high risk of recurrence, roughly 20% of individuals (5 out of 22 patients from Chinese cohort and 40 out of 245 patients from TCGA, respectively) exhibited MAGI3 expression at a moderate level (protein H-score between 4 ~ 6 or mRNA between 200 ~ 261.5 RPKM). Notably, our study suggests that ccRCC patients with MAGI3 levels in the middle to high range (protein H-score ≥ 4 or mRNA level >200 RPKM) may derive substantial benefits from Sunitinib treatment. Therefore, it is proposed that about 20% of ccRCC patients in stage I with MAGI3 exhibited in middle levels (H-score = 4 ~ 6 or mRNA between 200 ~ 261.5 RPKM) may benefit from Sunitinib adjuvant therapy. This collective evidence underscores the potential of MAGI3 as a predictive marker for tailoring treatment in early-stage ccRCC patients. However, further verification is necessary.

Based on the primary findings of this study, we have also preliminarily explored the potential of MAGI3 as a prognostic biomarker in other types of renal cancer, such as kidney renal papillary cell carcinoma and chromophobe renal cell carcinoma. Prognostic analysis indicated that there were no significant differences in overall survival between patients with high and low MAGI3 expression in these types of renal cancers (Supplementary Fig. 4B). This highlights the specificity of MAGI3 in ccRCC. Furthermore, we are considering targeting MAGI3 to improve resistance to Sunitinib in ccRCC patients. An initial approach could involve screening for drugs that mimic the function of MAGI3-PDZ1, thereby achieving negative regulation of the MAS-ERK pathway to address Sunitinib resistance in ccRCC patients. This strategy underscores the therapeutic potential of targeting specific molecular interactions within the signaling pathways that contribute to drug resistance.

This study acknowledges certain limitations that should be considered when interpreting its findings. One significant limitation is the relatively small cohort of ccRCC patients analyzed, which might restrict our ability to definitively establish MAGI3 as a reliable predictor for Sunitinib sensitivity. The power of our findings is constrained not only by the size of the sample but also by the retrospective nature of the data, emphasizing the necessity for future prospective studies.

In summary, this study reveals that reduced MAGI3 expression in ccRCC patients activates the ERK signaling pathway, promoting cell proliferation and Sunitinib resistance. MAGI3 functions as a novel tumor suppressor in ccRCC, emphasizing the critical role of the MAGI3/MAS/ERK axis in Sunitinib resistance. Diminished MAGI3 levels are associated with an unfavorable prognosis, particularly in early-stage ccRCC, suggesting its potential as a valuable prognostic biomarker. This underscores the prospective use of MAGI3 as a predictive marker for personalized treatment in early-stage ccRCC, enhancing clinical decision-making.

## Supplementary information

supplementary figures

supplementary table 1

supplementary table 2

supplementary table 3

supplementary table 4

supplementary table 5

supplementary table 6

Supplementary figure and table legends

supplementary materials and methods

original data

## Data Availability

The datasets used and/or analyzed during the current study are available from the corresponding author upon reasonable request. The public datasets analyzed in this study were obtained from the following sources: TCGA (Kidney Renal Clear Cell Carcinoma, PanCancer Atlas), GSE53757, GSE16449, GSE66271 and GSE64052. Functional analysis of the DEGs was conducted using the DAVID bioinformatics database for pathway enrichment analysis (KEGG) (http://david.ncifcrf.gov/tools.jsp).
