# Supplementary material for: MAGI3 enhances sensitivity to sunitinib in renal cell carcinoma by suppressing the MAS/ERK axis and serves as a prognostic marker
Source: Cell Death Dis. 2025 Feb 16;16(1):102. doi: 10.1038/s41419-025-07427-0 (PMC11830799; doi:10.1038/s41419-025-07427-0)
Supplement: Supplementary file 2 — supplementary table 1 [file 41419_2025_7427_MOESM2_ESM.doc]

Supplemental Table 1. Analysis of MAGI3 and clinical pathological factors in patients

with stage Ⅰ-Ⅳ ccRCC in TCGA database who did not receive adjuvant therapy

|  | Low MAGI3  (RPKM≤261.5) | High MAGI3  (RPKM>261.5) | X^2^ P Value |
| --- | --- | --- | --- |
| Variable | n=307 | n=157 |
| Age(year) |  |  |  |
| ≥60 | 149 | 64 | 2.53 0.11 |
| <60 | 158 | 93 |
| Gender |  |  |  |
| Male | 202 | 89 | 3.69 0.054 |
| Female | 105 | 68 |
| Grade |  |  |  |
| G1-2 | 166 | 89 | 0.64 0.42 |
| G3-4 | 138 | 63 |
