# Supplementary material for: MAGI3 enhances sensitivity to sunitinib in renal cell carcinoma by suppressing the MAS/ERK axis and serves as a prognostic marker
Source: Cell Death Dis. 2025 Feb 16;16(1):102. doi: 10.1038/s41419-025-07427-0 (PMC11830799; doi:10.1038/s41419-025-07427-0)
Supplement: Supplementary file 3 — supplementary table 2 [file 41419_2025_7427_MOESM3_ESM.doc]

Supplemental Table 2. Analysis of MAGI3 and clinical pathological factors in patients

with stageⅠccRCC in TCGA database who did not receive adjuvant therapy

|  | Low MAGI3  (RPKM≤261.5) | High MAGI3  (RPKM>261.5) | X^2^ P Value |
| --- | --- | --- | --- |
| Variable | n=146 | n=99 |
| Age(year) |  |  |  |
| ≥60 | 69 | 59 | 3.60 0.06 |
| <60 | 77 | 40 |
| Gender |  |  |  |
| Male | 89 | 54 | 0.99 0.32 |
| Female | 57 | 45 |
| Grade |  |  |  |
| G1-2 | 86 | 68 | 3.32 0.07 |
| G3-4 | 59 | 28 |
