# Supplementary material for: MAGI3 enhances sensitivity to sunitinib in renal cell carcinoma by suppressing the MAS/ERK axis and serves as a prognostic marker
Source: Cell Death Dis. 2025 Feb 16;16(1):102. doi: 10.1038/s41419-025-07427-0 (PMC11830799; doi:10.1038/s41419-025-07427-0)
Supplement: Supplementary file 4 — supplementary table 3 [file 41419_2025_7427_MOESM4_ESM.docx]

Supplementary Table 3. Univariate and multivariate Cox regression analyses of potential poor prognostic factors in stage I clear cell renal cell carcinoma

|  | Univariate |  |  | Multivariate |  |
| --- | --- | --- | --- | --- | --- |
| Variable | RR (95%CI) | *P* Value |  | RR (95%CI) | *P* Value |
| Age(year) |  |  |  |  |  |
| ≤60 | 2.895 (0.766 to 10.940) | 0.117 |  | 0.371 (0.096 to 1.440) | 0.152 |
| >60 | 1 |  |  | 1 |  |
| Gender |  |  |  |  |  |
| Female | 0.325 (0.042 to 2.541) | 0.284 |  | 0.432 (0.051 to 3.664) | 0.442 |
| Male | 1 |  |  | 1 |  |
| Grade |  |  |  |  |  |
| I | 1.053 (0.321 to 3.454) | 0.933 |  | 0.705 (0.199 to 2.500) | 0.588 |
| Ⅱ-Ⅲ | 1 |  |  | 1 |  |
| MAGI3 H-score |  |  |  |  |  |
| Low | 5.975 (1.642 to 26.129) | 0.035 |  | 5.261 (1.065 to 25.997) | 0.042 |
| High | 1 |  |  | 1 |  |
