# Supplementary material for: MAGI3 enhances sensitivity to sunitinib in renal cell carcinoma by suppressing the MAS/ERK axis and serves as a prognostic marker
Source: Cell Death Dis. 2025 Feb 16;16(1):102. doi: 10.1038/s41419-025-07427-0 (PMC11830799; doi:10.1038/s41419-025-07427-0)
Supplement: Supplementary file 5 — supplementary table 4 [file 41419_2025_7427_MOESM5_ESM.docx]

Supplemental Table 4. Docking energy table between MAGI3-PDZ1 domain

and MAS carboxy terminal ETVV sequence

| Position in peptide ETVV | Amino acid | Amino acids interact with ETVV peptide in the PDZ1 domain | Binding free energies（kcal/mol） |
| --- | --- | --- | --- |
| 0 | Valine, V | Glycine, G | -3.7 |
| -1 | Valine, V | Phenylalanine, F | -5.8 |
| -2 | Threonine, T | Isoleucine, I | -7.1 |
| -3 | Glutamate, E | Threonine, T | -4.2 |
