# Supplementary material for: MAGI3 enhances sensitivity to sunitinib in renal cell carcinoma by suppressing the MAS/ERK axis and serves as a prognostic marker
Source: Cell Death Dis. 2025 Feb 16;16(1):102. doi: 10.1038/s41419-025-07427-0 (PMC11830799; doi:10.1038/s41419-025-07427-0)
Supplement: Supplementary file 6 — supplementary table 5 [file 41419_2025_7427_MOESM6_ESM.docx]

| Protein | ICL2 Sequence | | | | | | | | |
| --- | --- | --- | --- | --- | --- | --- | --- | --- | --- |
| MRGPRX2 (134-142) | P | I | W | Y | R | C | R | R | P |
| MAS (137-145) | P | I | W | Y | R | C | H | R | P |

Supplemental Table 5. Multi-sequence alignment of the intracellular loop 2 (ICL2)

of MRGPRX2 and MAS
