# Supplementary material for: MAGI3 enhances sensitivity to sunitinib in renal cell carcinoma by suppressing the MAS/ERK axis and serves as a prognostic marker
Source: Cell Death Dis. 2025 Feb 16;16(1):102. doi: 10.1038/s41419-025-07427-0 (PMC11830799; doi:10.1038/s41419-025-07427-0)
Supplement: Supplementary file 7 — supplementary table 6 [file 41419_2025_7427_MOESM7_ESM.doc]

Supplemental Table 6. Analysis of MAGI3 and clinical pathological factors in ccRCC patients

in TCGA database who received Sunitinib therapy

|  | Low MAGI3  (RPKM≤200) | High MAGI3  (RPKM>200) | X^2^ P Value |
| --- | --- | --- | --- |
| Variable | n=37 | n=11 |
| Age(year) |  |  |  |
| ≥60 | 15 | 6 | 3.60 0.06 |
| <60 | 22 | 5 |
| Gender |  |  |  |
| Male | 27 | 9 | 0.35 0.55 |
| Female | 10 | 2 |
| Grade |  |  |  |
| G1-2 | 8 | 5 | 2.44 0.12 |
| G3-4 | 29 | 6 |
