# Supplementary material for: MAGI3 enhances sensitivity to sunitinib in renal cell carcinoma by suppressing the MAS/ERK axis and serves as a prognostic marker
Source: Cell Death Dis. 2025 Feb 16;16(1):102. doi: 10.1038/s41419-025-07427-0 (PMC11830799; doi:10.1038/s41419-025-07427-0)
Supplement: Supplementary file 8 — Supplementary figure and table legends [file 41419_2025_7427_MOESM8_ESM.docx]

**Supplementary figure and table legends**

**Suppl. Figure 1. MAGI3 emerges as a novel prognostic marker in ccRCC and participates in MAPK signaling.**

**A.** Venn diagram illustrating common DEGs identified in four independent ccRCC cohorts through mRNA expression profiling of tumor and normal renal tissue.

**B.** KEGG pathway enrichment analysis of the DEGs using the DAVID bioinformatics database.

**C.** Hazard ration of MAPK-related genes based on mRNA expression in ccRCC patients from TCGA analyzed for overall survival (OS).

**D, E.** Comparison of MAGI3 mRNA expression levels in ccRCC specimens to normal tissues from GSE16449 (D) and GSE53757 (E).

**F.** Evaluation of MAGI3 mRNA expression levels in ccRCC specimens compared to paired normal tissues from GSE66271.

Data presented as mean ± SEM, with statistical significance calculated using the paired t-test. ***, P < 0.001.

**Suppl. Figure 2. The significant association of MAGI3 with tumor proliferation in ccRCC.**

**A.** GSEA enrichment plots exhibit significant activation of epithelial cell proliferation pathways in ccRCC specimens with middle-low (M/L) MAGI3 expression from the TCGA ccRCC dataset.

**B.** Tumor lengths were notably different between TCGA ccRCC patients with M/L and high MAGI3 expression, assessed through t-test analysis (****, P < 0.0001).

**C.** MAGI3 overexpression induces cell cycle arrest. Cell cycle arrest at the G1-S transition in 786-O cells is illustrated by flow cytometry assay following MAGI3 overexpression.

**Suppl. Figure 3. MAGI3 inhibits the activation of the ERK pathway through its interaction with MAS.**

**A.** GSEA enrichment plots reveal significant activation of ERK pathways in ccRCC specimens with middle-low (M/L) MAGI3 expression from the TCGA ccRCC dataset.

**B.** Quantification of pERK/GAPDH in Figure 4A.

**C.** Quantification of pERK/GAPDH in Figure 4B.

**D.** Overexpression of MAGI3 reduced phospho-ERK levels in cells transfected with the MAS WT but not the MAS V325A mutant.

Data is presented as mean ± SEM, with statistical significance determined using an unpaired t-test. ** indicates significance at P < 0.01; NS indicates non-significance.

**Suppl. Figure 4. MAGI3 is downregulated in Sunitinib-resistant renal clear cell carcinoma cell lines, and has no prognostic significance for other subtypes of renal cancer patients.**

**A.** Significant downregulation of MAGI3 mRNA in sunitinib-resistant ccRCC 786-O cell line (GSE64052). Mean ± SEM. Two-tailed unpaired t-test. ***p < 0.001.

**B.** KM survival plots for overall survival (OS) based on MAGI3 mRNA expression in kidney renal papillary cell carcinoma (KIRP) and Kidney Chromophobe (KICH) patients from TCGA dataset (p > 0.05, log-rank test).

Supplemental Table 1. Analysis of MAGI3 and clinical pathological factors in patients with stage Ⅰ-Ⅳ ccRCC in TCGA database who did not receive adjuvant therapy

Supplemental Table 2. Analysis of MAGI3 and clinical pathological factors in patients with stageⅠccRCC in TCGA database who did not receive adjuvant therapy

Supplementary Table 3. Univariate and multivariate Cox regression analyses of potential poor prognostic factors in stage I clear cell renal cell carcinoma

Supplemental Table 4. Docking energy table between MAGI3-PDZ1 domain and MAS carboxy terminal ETVV sequence

Supplemental Table 5. Multi-sequence alignment of the intracellular loop 2 (ICL2) of MRGPRX2 and MAS

Supplemental Table 6. Analysis of MAGI3 and clinical pathological factors in ccRCC patients

in TCGA database who received Sunitinib therapy
