# Supplementary material for: MAGI3 enhances sensitivity to sunitinib in renal cell carcinoma by suppressing the MAS/ERK axis and serves as a prognostic marker
Source: Cell Death Dis. 2025 Feb 16;16(1):102. doi: 10.1038/s41419-025-07427-0 (PMC11830799; doi:10.1038/s41419-025-07427-0)
Supplement: Supplementary file 9 — supplementary materials and methods [file 41419_2025_7427_MOESM9_ESM.docx]

**Materials and Methods**

**Antibodies and Reagents**

The antibodies utilized in this study were procured from the following suppliers: anti-Flag antibody (Sigma-Aldrich, St. Louis, MO), anti-GFP and anti-His antibodies (D291-3S, MBL, Nagoya, Japan), anti-MAGI3 and anti-MAS antibodies (NBP1-17210, NBP1-78444, NOVUS Biologicals, Littleton, CO), anti-MAGI3 antibody for immunoprecipitation (IP) (sc-136471, Santa Cruz Biotechnology, Dallas, TX), anti-β-actin antibody (bs-0061R, Bioss, Beijing, China), anti-GAPDH antibodies (bsm-33033M, Bioss, Beijing, China), anti-GST antibody (GE Healthcare, Buckinghamshire, UK), anti-ERK and anti-pERK antibodies (#9102, #4370, Cell Signaling Technology), anti-Ki67 antibody (ZM-0166, ZSGB-BIO, Beijing, China), and anti-pERK antibody for immunohistochemistry (IHC) (GB11507-100, ServiceBio, Wuhan, China). HRP-conjugated secondary antibodies were additionally obtained from ZSGB-BIO (Beijing, China).

Additionally, the study utilized reagents including: Ang-(1-7) (586962-44-9, BACHEM, Torrance, CA), A779 (HY-P0216, MedChemExpress, Shanghai, China), SCH772984 (SCH) (HY-50846, MedChemExpress, Shanghai, China), Sunitinib (HY-10255A, MedChemExpress, Shanghai, China), CCK-8 (Dojindo, Kumamoto, Japan), glutathione-agarose beads (Sigma-Aldrich, St. Louis, MO), protein A/G-agarose (Santa Cruz Biotechnology), Matrigel (Corning, New York) and Hematoxylin eosin (HE) staining kit (Beyotime, Shanghai, China).

**Cell Lines, Transfection, and Lentiviral Infection**

COS-7 and BHK cells were acquired from the Institute of Basic Medical Sciences (Beijing, China), and human clear cell renal cell carcinoma cell lines (786-O and 769-P) were obtained from Procell Life Science & Technology (Wuhan, China). These cells were cultured in DMEM (COS-7), MEM (BHK), or RPMI-1640 (786-O and 769-P) medium with 10% FBS and 1% penicillin/streptomycin at 37°C with 5% CO_2_. Prior to use, cells were authenticated using short tandem repeat (STR) DNA fingerprinting and tested negative for mycoplasma contamination. Passage number was limited to 30 continuous passages.

For transient transfection, Lipofectamine 3000 (Invitrogen, Carlsbad, CA) was employed following the manufacturer's guidelines. MAGI3 cDNA was integrated into the lentiviral vector prLV-puro, and lentivirus was packaged by Likely (Beijing, China). Lentiviral particles carrying MAGI3 shRNA were obtained from Santa Cruz Biotechnology. Stable cell lines expressing MAGI3 cDNA or MAGI3 shRNA were established through lentiviral infection with 10 μg/mL polybrene (Santa Cruz Biotechnology), followed by selection with 2 μg/mL puromycin (Solarbio, Beijing, China) for 7 days. Confirmation of MAGI3 overexpression or knockdown was performed through immunoblotting.

**Cell Viability Assay and Cell Cycle Analysis**

Cells, seeded at a density of 1500 cells per well in 96-well microplates, adhered and were cultured in a serum-deprived medium with 1% FBS and 1 μM Ang-(1-7). Following the specified incubation, CCK-8 reagent was added, and after an additional 1-hour incubation, cell viability was measured at 450 nm using an EnVision multilabel reader (PerkinElmer, Waltham, MA).

For cell cycle analysis, cells were fixed in 70% ethanol at 4 °C overnight, washed with PBS, stained with propidium iodide for 30 minutes, and analyzed using flow cytometry (EPICS@XL, Beckman Coulter, 250 S. Kraemer Boulevard Brea, CA). In certain experiments, cells were pretreated with selective inhibitors for 30 minutes before Ang-(1-7) treatment.

**Colony Formation Assay**

One hundred cells (eight hundred cells in Sunitinib drug sensitivity experiments) were seeded in 1 mL of complete medium in a six-well plate and allowed to adhere. Cells were then cultured with or without Ang-(1-7)/Sunitinib for 7-10 days. After three PBS washes, cells were fixed with 4% paraformaldehyde, stained with crystal violet, and colonies with more than 50 cells were counted using an inverted microscope. In inhibitor experiments, A779 and SCH were added 30 minutes before Ang-(1-7) treatment.

**GST Pull-Down,** **Immunoprecipitation (IP) and Immunoblotting (IB)**

We performed protein purification (GST- or His-fused proteins), GST pull-down assays, cell lysis, immunoprecipitation (IP), and immunoblotting (IB) following established protocols ^[1, 2]^. For ERK signaling studies, cells were seeded in six-well plates, adhered for 24 hours, underwent 24-hour serum starvation, and were then stimulated with Ang-(1-7) for the indicated timeframe. In select experiments, cells were pre-treated with inhibitors for 30 minutes before Ang-(1-7) addition.

**Plasmids and siRNA**

Plasmids expressing HA-tagged Mas, GFP-tagged MAGI3, and V5/His-tagged MAGI3 were kindly provided by Dr. Randy Hall (Emory University, GA). Additionally, previously described plasmids containing PDZ domains of MAGI3 (pET30A) and Mas-CT or its mutant (pGEX-4T-1) were utilized ^[3]^. Wild-type and V325A mutant Mas plasmids in pEGFP-C2 were created via PCR amplification and confirmed through bidirectional sequencing.

siRNAs were obtained from Sango Biotech (Shanghai, China). The MAGI3 siRNA was designed to target the sequence 5’-GGUCCACCAUCAGGAACAAACUCAG-3’. The control siRNA sequence was 5’-UUCUCCGAACGUGUCACGUTT-3’.

**Overlay Assay**

To assess the GST-Mas-CT fusion protein's interaction with MAGI3 PDZ domains, we utilized a blot overlay technique. The His-tagged fusion proteins were separated on SDS-PAGE, transferred onto membranes, and overlaid with purified GST-Mas-CT (80 nM) in a blot buffer (2% milk, 0.1% Tween 20 in PBS). The overnight incubation at 4°C was followed by probing with an anti-GST antibody, and signal visualization using enhanced chemiluminescence.

**Immunofluorescence**

BHK cells in 6-well plates were transfected with GFP-Mas and Flag-MAGI3, followed by fixation, permeabilization, and co-localization assessment using anti-Flag and anti-GFP antibodies. Visualization was achieved through confocal microscopy (Leica DM6000 CS, Germany) after secondary antibody incubation and DAPI staining for nucleus visualization.

**IHC Staining**

Xenograft tumors were fixed in 4% paraformaldehyde, paraffin-embedded, and sectioned into 6 μm-thick slices using a Leica microtome. After dewaxing, antigen retrieval was performed in a 10 mM sodium citrate solution (pH 6.0), followed by standard hematoxylin and eosin staining.

For immunohistochemistry (IHC), sections were incubated with primary antibodies overnight at 4°C, followed by treatment with HRP-conjugated secondary antibodies for 15 minutes. Signal visualization utilized a DAB detection kit (ZSGB-BIO), and counterstaining was performed with haematoxylin (Solarbio). After dehydration, slides were sealed with resin and scanned using an Aperio GT450 digital slide scanner, with images captured using ImageScope software.

**Bioinformatics**

In Bioinformatics, TCGA ccRCC RNA-seq and clinical data, along with GEO ccRCC microarray data from Synapse (https://www.synapse.org/) and cBioPortal (http://www.cbioportal.org/) were obtained. GEPIA2 (http://gepia2.cancer-pku.cn/) and GEO2R identified differentially expressed genes (DEGs) in ccRCC with a fold change > 2 or < -2. Venn diagrams were generated using the Venny website (https://bioinfogp.cnb.csic.es/tools/venny/), and DAVID (https://david.ncifcrf.gov/) performed functional annotations of DEGs. Gene Set Enrichment Analysis (GSEA) with default parameters and 1000 permutations assessed statistical significance of enrichment from the Molecular Signatures Database (http://www.gsea-msigdb.org/), considering enrichment significant if the false discovery rate was < 0.25 and P < 0.05.

**Protein Complex Prediction Using AlphaFold**

AlphaFold-predicted structures for MAGI3 and MAS were obtained from the AlphaFold Protein Structure Database, and subsequent molecular docking simulations revealed interaction dynamics between MAGI3's PDZ1 domain and MAS's carboxyl-terminal segment. The Protein Data Bank supplied the structural model for MRGPRX2 and G proteins (PDB: 7S8N). Structural figures were created using the PyMOL program (<http://pymol.sourceforge.net/>).

**Reference**

[1] Zheng S, Yang Y, Song R, Yang X, Liu H, Ma Q, et al. Ang-(1-7) promotes the migration and invasion of human renal cell carcinoma cells via Mas-mediated AKT signaling pathway. Biochem Biophys Res Commun 2015; 460: 333-40.10.1016/j.bbrc.2015.03.035.

[2] Yang X, Zheng J, Xiong Y, Shen H, Sun L, Huang Y, et al. Beta-2 adrenergic receptor mediated ERK activation is regulated by interaction with MAGI-3. Febs Lett 2010; 584: 2207-12.10.1016/j.febslet.2010.03.039.

[3] He J, Bellini M, Inuzuka H, Xu J, Xiong Y, Yang X, et al. Proteomic Analysis of β1-Adrenergic Receptor Interactions with PDZ Scaffold Proteins. J Biol Chem 2006; 281: 2820-7.10.1074/jbc.M509503200.
