# Supplementary figures and images for: MAGI3 enhances sensitivity to sunitinib in renal cell carcinoma by suppressing the MAS/ERK axis and serves as a prognostic marker
Source: Cell Death Dis. 2025 Feb 16;16(1):102. doi: 10.1038/s41419-025-07427-0 (PMC11830799; doi:10.1038/s41419-025-07427-0)

Figure 2

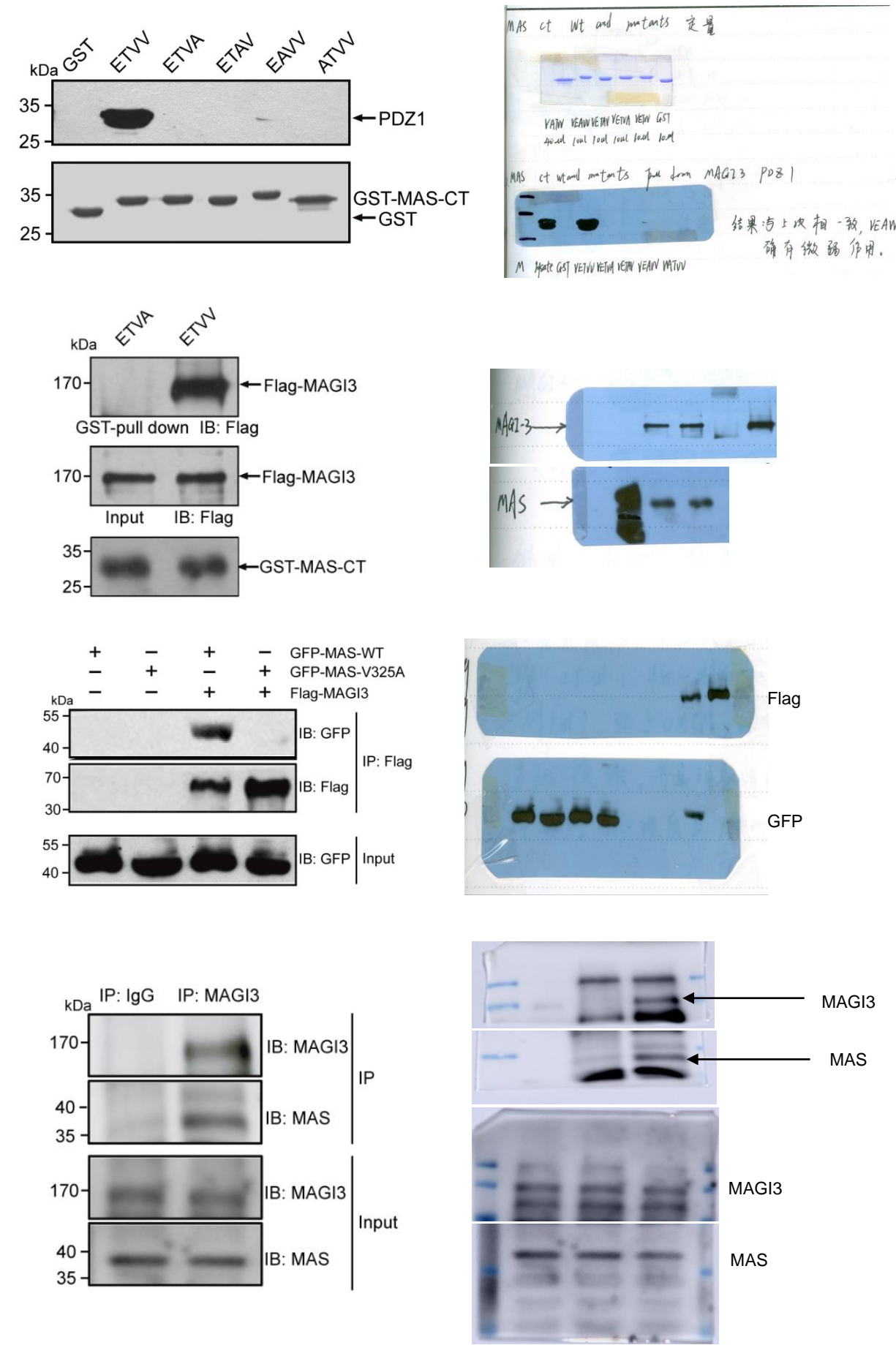

Figure 3

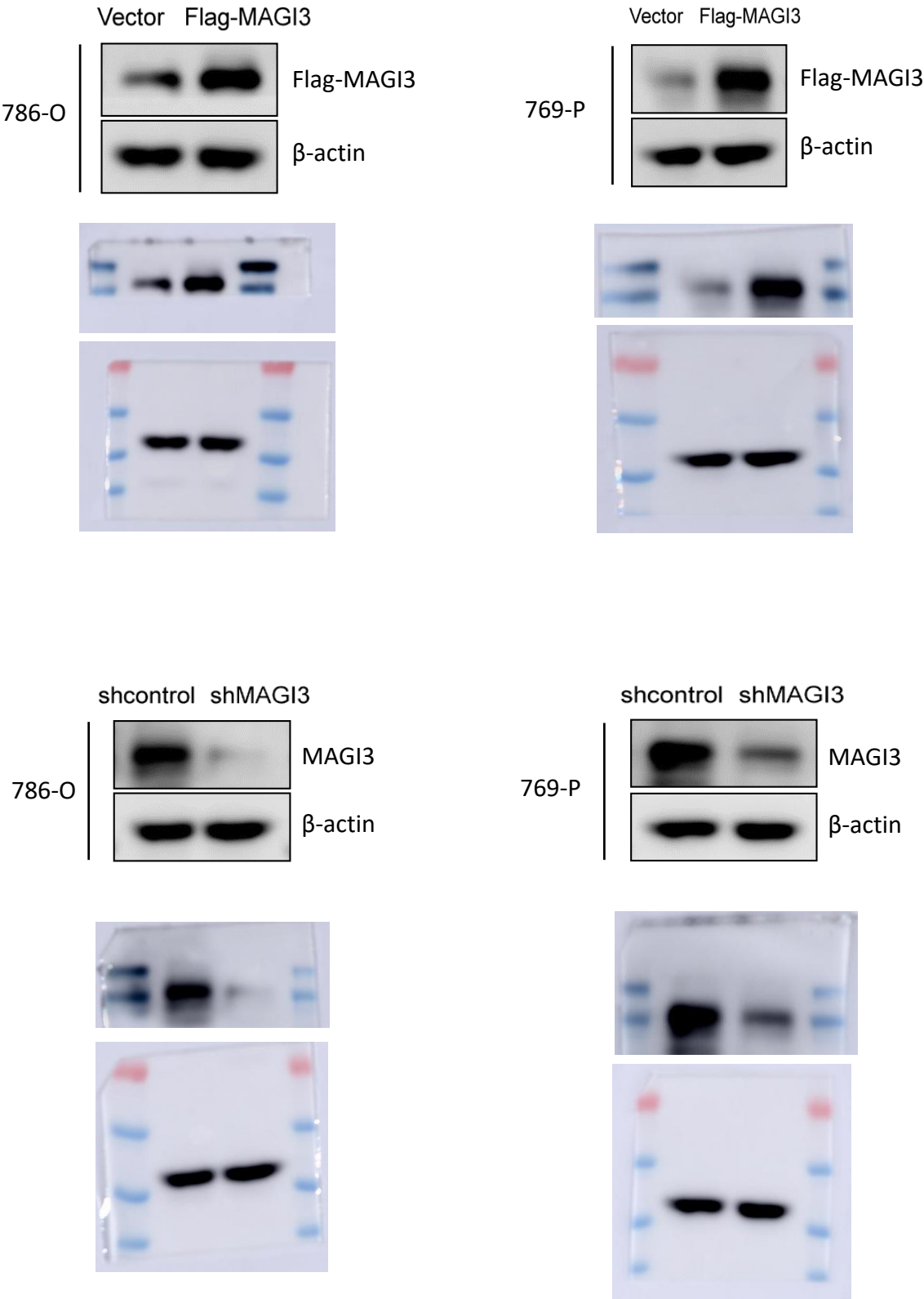

Figure 4

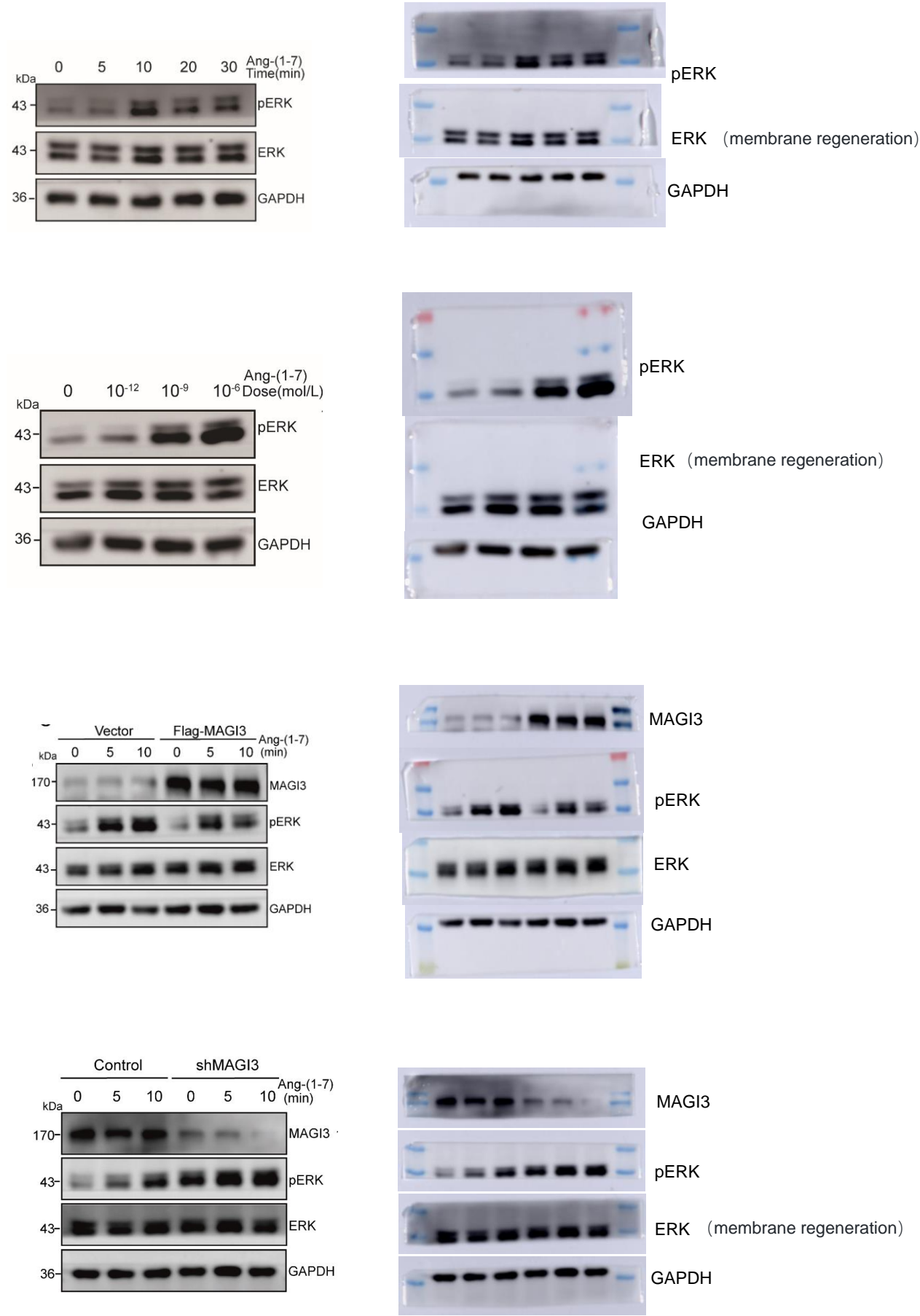

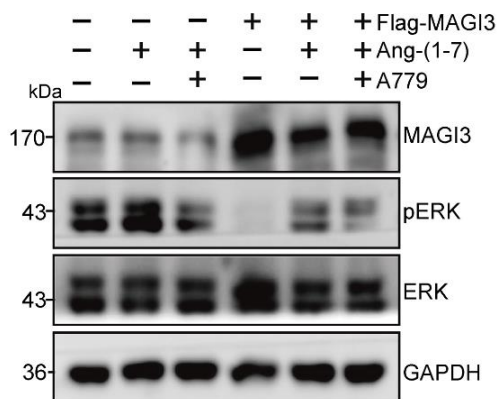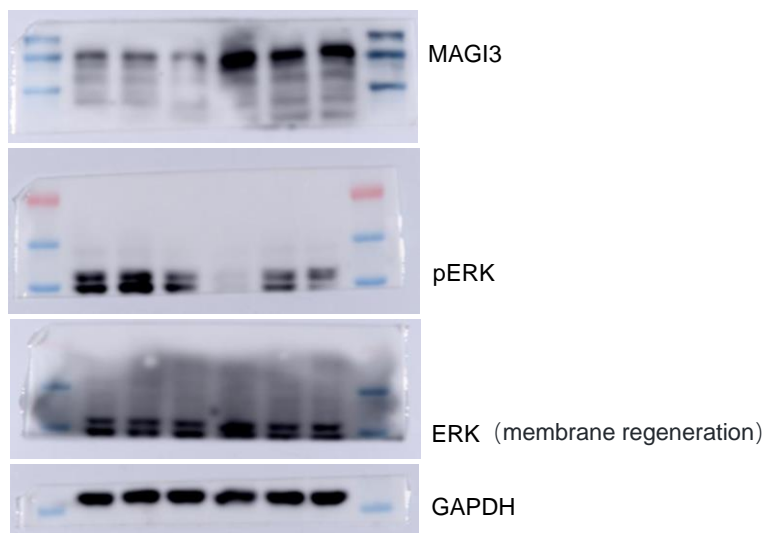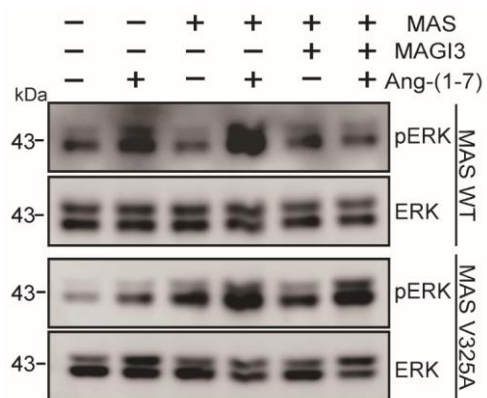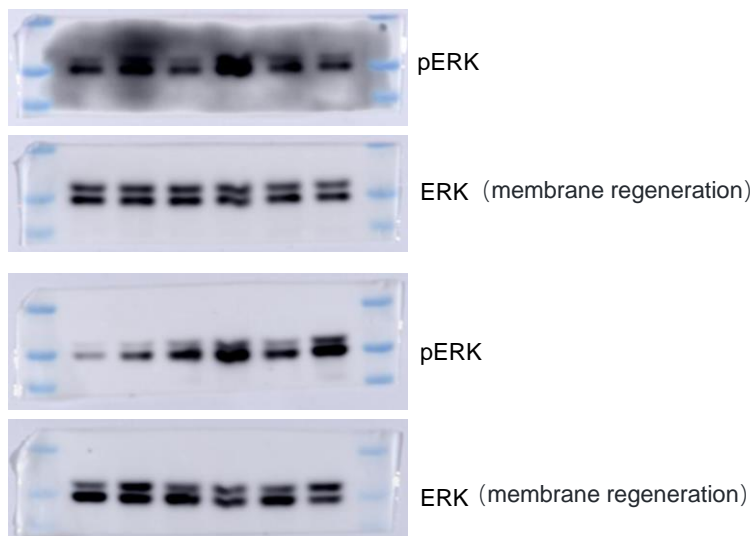

Supplement: Supplementary file 10 — original data [file 41419_2025_7427_MOESM10_ESM.pdf]
